# Review of Emerging Strategies and Progress in Transition Metal-Modified Activated Carbons for Hazardous Gas Elimination

**DOI:** 10.1021/acsomega.5c12100

**Published:** 2026-03-19

**Authors:** Yue Wang, Yanle Pei, Yihao Zhang, Chaoqun Li, Zhen Song, Huichuang Guo, Hailong Shu

**Affiliations:** State Key Laboratory of Chemistry for NBC Hazards Protection, Beijing 102205, China

## Abstract

Hazardous gas emissions, including volatile organic compounds (VOCs), nitrogen oxides (NO_*x*_), sulfur dioxide (SO_2_), elemental mercury (Hg^0^), carbon dioxide (CO_2_), and chemical warfare agents (CWAs), pose severe threats to human health and the environment, driving the need for efficient, cost-effective removal technologies. Activated carbon (AC), renowned for its high surface area, tunable porosity, and economic viability, serves as an ideal support for transition metal modification (e.g., Mn, Fe, Co, Ni, Cu, Zn), which imparts enhanced catalytic activity and selectivity through redox and acid–base functionalities. This review systematically summarizes recent advances in transition metal-modified ACs for hazardous gas elimination, covering preparation methodologies (impregnation, doping, sol–gel, and composites), adsorption mechanisms (physisorption vs chemisorption, and diffusion processes), key influencing factors (pore structure, surface chemistry, metal dispersion, gas properties, and operational conditions), and practical applications across diverse pollutants. Key insights highlight the synergistic roles of metal loading in bridging physical adsorption and catalytic conversion, while addressing challenges such as pore blockage, humidity interference, and multipollutant competition. Future perspectives prioritize operando mechanistic studies, scalable engineering processes, and precision synthesis to bridge the gap between fundamental research and industrial application, achieving scalable, high-performance solutions for real-world environmental remediation.

## Graphical abstract

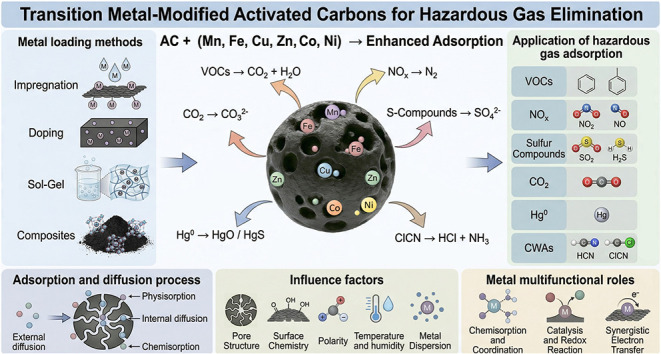

## 1 Introduction

Modern society’s fast pace has turned harmful gas emissions into a pressing worldwide issue, damaging ecosystems, risking human health, and hindering sustainable development. Among the most concerning pollutants are volatile organic compounds (VOCs, such as aromatic types), mercury, nitrogen oxides (NO_*x*_), sulfur-based gases, carbon dioxide (CO_2_), and chemical warfare agents like chlorine cyanide.^1^ As shown in Figure 1, these harmful gases come from both natural and human activities. In cities, much of the NO_*x*_ ^2^ and VOCs^3^ result from extracting, storing, refining, moving, and burning fossil fuels. Materials used in home decoration often release formaldehyde and benzene-based vapors. Coal power stations produce large amounts of sulfur dioxide, while farming—through fertilizers and animal waste—releases ammonia and nitrous oxide. Industrial activities, such as making cement, producing steel, and smelting metals, add further to CO_2_ levels and send heavy metals like mercury and arsenic into the air.^4^ The impact of these substances is serious: even small amounts of many VOCs—especially aromatic types—can be foul-smelling, toxic, and carcinogenic, harming the respiratory tract, skin, the nervous system, and blood quality.^5^ Ammonia can cause breathing problems, acidify soil, and overenrich water bodies. Nitrogen oxides contribute to acid rain and urban smog, and can weaken immunity and heart function. Sulfur dioxide damages buildings and forests; CO_2_ drives global warming and more extreme weather; chemical agents pose security risks; and sulfides worsen air quality and lung health. This makes creating effective ways to remove such gases an urgent priority.

**Figure 1:**
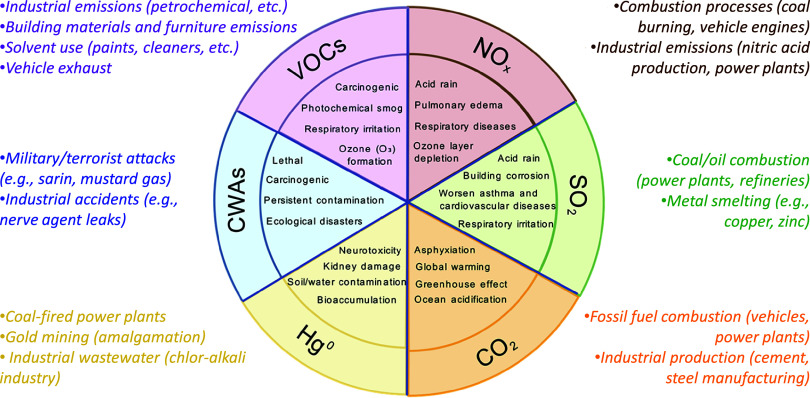
Sources and hazards of typical hazardous gases.

Traditional methods for eliminating hazardous gases can be divided into two main categories. The first includes destructive techniques, such as catalytic oxidation, which completely convert pollutants into harmless substances.^6^ The second encompasses recovery techniques—including absorption, adsorption, filtration, condensation, and membrane separation—that capture gases by modifying thermodynamic conditions. The effectiveness of these methods is often enhanced by incorporating transition metals, which either participate directly in chemical reactions or act as catalysts to accelerate the elimination process. Recent advances highlight the potential of metal-doped novel materials, including carbonaceous substrates, metal–organic frameworks (MOFs),^7^ zeolites,^8^ hyper-cross-linked polymers (HCPs),^9^ transition metal nanoparticles,^10^ bimetallic catalysts,^11^ metal oxides,^12^ and metallic nanosheets^13^ for applications in industrial flue gas treatment, air purification, VOC abatement, pharmaceuticals, and food processing. Synergistic material design enables efficient, economical, and environmentally sound elimination, meeting stringent environmental standards across complex scenarios.

AC remains a cornerstone material for eliminating multiphase pollutants (gaseous and aqueous) owing to its tunable porosity, high surface area, favorable textural properties, abundant precursor availability, mature industrial production and low cost. Continuous technological refinements further enhance its economic viability. ACs are usually derived from carbon-rich materials by carbonization and activation, forming a graphitic microcrystalline structure. They can be classified into coal/petroleum-coke-based, polymer-based and biomass-based (classified as biochar in some literatures) variants. Its surface functionality, acid–base/redox tunability and stability in different media render it an ideal supporter for metal modification.^14^ What’s more, cost-effectiveness and precursor accessibility solidify its status as a benchmark adsorbent still active today. Surface architecture of porous carbon is illustrated in Figure 2.

**Figure 2:**
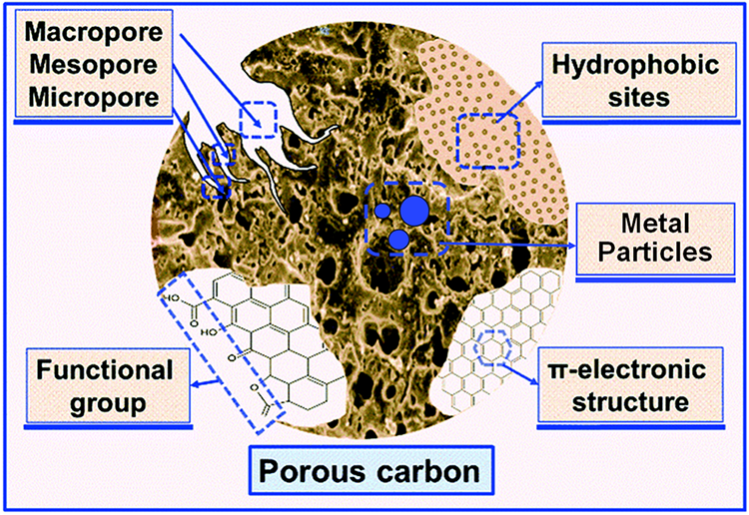
Surface architecture of porous carbon. (Reprinted with permission from ref 15, Copyright 2022, Royal Society of Chemistry).

Transition metals (e.g., Mn, Fe, Co, Ni, Cu, and Zn) exhibit distinctive electronic configurations and reactivity profiles, primarily attributed to the versatile energetics, symmetry characteristics, and electron accommodation capacity of their partially occupied d-orbitals. These metals typically have up to nine valence orbitals arising from the (*n*–1) d, ns, and np subshells, enabling them to form multiple bonds and facilitate electron transfer. These fundamental properties empower them to simultaneously stabilize adsorbed molecular species and activate substrates through electron transfer processes, thereby serving as pivotal active centers in high-performance adsorbents and catalytic systems. Transition metals predominantly exist as oxides or sulfides in the Earth’s crust, ensuring wide availability, low-cost extraction, and economical production. Their diverse chemical forms allow versatile integration into various material systems. The reactivity of transition metals can be precisely modulated through oxidation state variation, elemental compositing, and doping strategies. Therefore, they can be loaded on ACs to enhance their performance in selective adsorption and efficient elimination of targeted hazardous gases.

Recent review articles have comprehensively summarized the applications of carbon-based materials in environmental remediation,^15^ the adsorption–desorption mechanisms of VOCs on porous carbon adsorbents,^16^ as well as the elimination of heavy metal elements from both atmospheric and aqueous environments using transition metal catalysts.^17^ However, these studies either fail to provide dedicated discussions on the specific roles of metallic components or indiscriminately combine gas-phase and liquid-phase adsorption phenomena without systematic differentiation. This review summarizes the systematic knowledge and recent advances in transition metal-loaded AC systems for hazardous gas removal. First, we overview the sources, toxicity, and environmental impacts of common hazardous gases. Next, we examine the characteristics and advantages of AC, followed by a summary of typical methods for preparing transition metal–supported adsorbents on AC. The adsorption processes, mechanisms, and key influencing factors under metal modification are analyzed. Then, progress in adsorption applications is reviewed according to gas types, covering both conventional and emerging approaches. Finally, we discuss current challenges and prospects of transition metal-modified AC systems for hazardous gas elimination.

## 2 Transition Metal Loading Methods on AC

Common methods for metal loading include impregnation, sol–gel, and doping methods, which are primarily differentiated by the specific stage of AC preparation when the metals are introduced, as graphically summarized in Figure 3.^18^ Typically, metal loading performed in the final step after carbonization and activation is classified as impregnation, while more thorough incorporation during the carbonization process is categorized as doping or sol–gel methods. Another approach is classified as composite materials, which involves combining AC with another adsorptive material to create synergistic effects. The metal may be loaded onto one or both components—either during their individual preparation or after the composite is formed.

**Figure 3:**
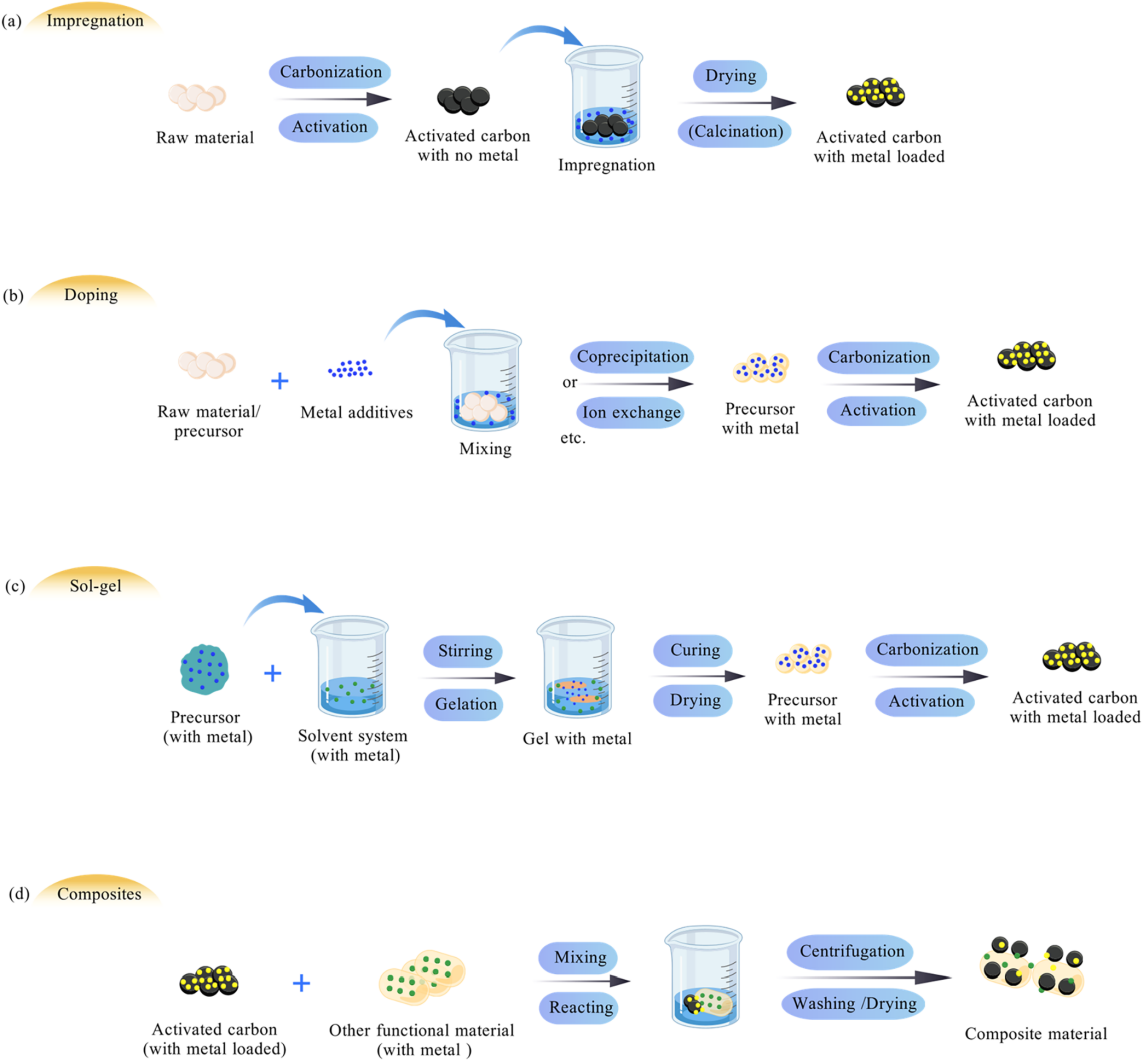
Schematic of metal-doped AC production (a) Impregnation; (b) Doping; (c) Sol–gel; (d) Composites.

These methodological variations critically influence: (i) the binding configuration and strength between metal species and carbon surfaces, (ii) the dispersion uniformity and spatial distribution of metal components, and (iii) the resultant efficacy of metal participation in adsorption reactions. Metal precursors may be introduced in various forms, including metallic oxides, salts, or elemental states (single atoms or nanoparticles). Several typical methods are introduced below.

### 2.1 Impregnation

The impregnation method remains the most widely used technique for loading metals onto AC, owing to its simplicity, low cost, and ease of operation. The standard procedure involves first obtaining pristine AC, then immersing it in a solution containing metal salts. The mixture is stirred to allow uniform distribution of metal ions throughout the carbon pores. Subsequently, the material is dried, often followed by calcination to firmly anchor the metal species onto the AC. During this step, metal ions are typically converted into metallic or metal oxide forms. Commonly used additives include hydroxides, carbonates, chromates, and nitrates.

In a representative study, Lu et al.^19^ prepared metal-impregnated carbons with 3 wt % loading by immersing coconut shell-derived AC in aqueous solutions of copper, cobalt, iron, and nickel nitrates. After processing, the resulting materials were tested for toluene removal and NO reduction, demonstrating enhanced catalytic activity due to uniform metal dispersion. Similarly, Lahuri et al.^20^ incorporated metal oxides such as CeO_2_, ZnO, and Co_3_O_4_ into AC to assess CO_2_ capture performance. In subsequent work, the same group^21^ further modified AC using ferrous sulfate solutions of different concentrations, obtaining iron oxide-loaded carbons with improved CO_2_ adsorption capacity. Pretreating the carbon with KMnO_4_ was found to increase surface oxygen functional groups, thereby enhancing interactions with the subsequently deposited iron oxides. In another example, Xiang et al.^22^ used solutions of CoCl_2_ or MnCl_2_ to prepare cobalt- or manganese-loaded ACs through impregnation, followed by drying and calcination, for elemental mercury adsorption from simulated flue gas.

The adsorption capacity of the metal-loaded samples has been specifically improved. This enhancement occurs primarily through two mechanisms: the impregnated metal oxides can directly react with the target adsorbates, promoting chemical adsorption; alternatively, the metal ions can modify the surface functional groups, thereby strengthening the chemisorption capability. While the impregnation method offers advantages such as lower cost and simpler operation compared to other techniques, it may also negatively alter the pore structure and compromise the cycling stability of the AC. Because the deposited metal species, after thermal treatment should have functioned in the form of dispersed nanoparticles like oxides, would partially block pores and lead to a reduction in adsorption capacity if used excessively.

### 2.2 Doping

The doping method differs from impregnation in that metals are added to the precursor material before the steps of carbonization and activation, after which the mixture is subjected to carbonization. This approach enables the metals to be deeply embedded within the carbon structure, forming strong chemical bonds, and typically results in higher stability, better dispersion, more effective adsorption sites and special reactivity than impregnation. In addition, during the activation process, some metals contribute to pore expansion in the AC, thereby widening the pore structure and enhancing adsorption capacity. Moreover, some of the metals may be reduced to their zerovalent state in the activating steam, which modifies their chemical adsorption properties.

Depending on the raw materials and additives, common doping methods include coprecipitation, and iron-exchange resin processes.

Precursor impregnation is simpler than coprecipitation, essentially moving the impregnation step to before carbonization. This method involves soaking the AC raw material in a simple metal salt solution, followed by drying, to obtain the precursor. For instance, Wang et al.^23^ applied this method to fabricate copper-loaded AC with high uniformity by mixing walnut shells with a copper-ammonia solution before the thermal processing steps. Zhou et al. prepared AC/MO*_x_* (M = Mg, Zn, Cu, and Zr) by impregnation of a commercial AC with aqueous solutions of metal nitrates. After drying, the precursor was calcined at high temperature in a N_2_ atmosphere, during which the nitrate decomposed into metal oxides, yielding metal-loaded AC. In this study, metals were impregnated onto mature AC, but subsequently subjected to carbonization, making the process more than simple impregnation.

In the coprecipitation process, carbon precursors are added into a metal-containing solution. Then the pH is adjusted to precipitate the metal ions onto the precursor surface to gain a universe and solid loading. In a typical example, Jia et al.^24^ synthesized iron-based modified biochar codoped with multiple metals, including cerium, copper, cobalt, and manganese, during biomass pyrolysis for removing gaseous elemental mercury (Hg^0^). The synthesis involved dissolving metal salts in an acidic HCl solution, adding pretreated walnut shell biomass, and rapidly introducing 25 wt % ammonia to adjust the pH to 9. After completing precipitation under heated stirring, the solid was filtered, washed, and dried to obtain metal-loaded precursors, which were finally activated at 600 °C in a tube furnace. The resulting materials exhibited uniformly dispersed metals within the iron-based phase, forming stable surface oxide systems with synergistic effects that enhanced Hg^0^ removal efficiency by up to 13-fold compared to unmodified biochar.

The ion-exchange resin method prepares AC by using the resin as a precursor, loading specific metals through ion exchange, and then proceeding with carbonization and activation. For instance, Li et al.^25^ used a weakly acidic phenolic cation-exchange resin as the carbon precursor, producing spherical copper-loaded AC through Cu^2+^ exchange, followed by carbonization and CO_2_ activation. Wang et al.^26^ added metal salts directly during the sulfonation (precarbonization) step of resin microspheres, promoting the binding of metal ions with surface sulfonic groups on resin. After drying and subsequent carbonization and activation, spherical AC was obtained, exhibiting an average granule crush strength of 100 N and significantly enhanced adsorption capacity for ClCN in ambient moist air. This in situ integration approach, compared to conventional impregnation, proved highly effective in improving mechanical strength, reducing fragmentation, and enhancing metal dispersion.

Compared with the impregnation method, which can result in partial mesopore blockage, the doping technique offers a significant advantage by actively promoting pore development during carbonization through the action of certain metals. This method achieves highly uniform dispersion of metal ions, with typical particle sizes remaining below 10 nm, thereby effectively preventing their aggregation on the carbon surface. As a result, the metal species are distributed both uniformly and deeply within the carbon matrix. The subsequent high-temperature treatment firmly anchors these metal species into the carbon structure, which substantially improves their leaching resistance. This strong integration fosters enhanced interactions between the metal and carbon, potentially leading to the formation of composite structures incorporating surface functional groups. These characteristics collectively promote more effective synergistic actions during adsorption processes and support improved catalytic performance.

### 2.3 Sol–gel

Distinct from methods such as impregnation or doping, which typically start with solid raw materials (such as coal, pitch, fruit shells, or resins) to produce metal-loading AC, the sol–gel method employs metal-containing ester salt solutions as precursors. Through hydrolysis, polycondensation, and other cross-linking reactions, a sol is formed, which solidifies into a gel upon solvent removal by drying. Subsequent carbonization and activation processes yield AC, thus giving the technique its name: the sol–gel method.

The sol–gel method can be classified into several categories based on the materials and reactions involved in sol formation. Two methods usually used in AC preparation for gas adsorption are as follows.

First, metal alkoxides react with organic additives through hydrolysis and polycondensation (forming M–O–M bonds), leading to gelation. The organic groups from the alkoxides and organic solvents can also serve as carbon sources. In a study, Wang et al.^27^ employed this method to synthesize TiO_2_–AC for catalytic NO_2_ degradation. of the synthesis employed tetrabutyl titanate as the polycondensation precursor and diethanolamine as a gel stabilizer. After vigorous stirring, tetrabutyl titanate underwent hydrolysis and condensation, forming a transparent sol. Subsequent drying yielded a yellow translucent gelThermal treatment then decomposed and carbonized the organic components and after natural cooling, a black TiO_2_–AC powder was obtained. It acts as a dual-functional material, significantly boosting photocatalytic dye degradation and enabling highly sensitive room-temperature NO_2_ gas sensing by concentrating target molecules near active sites.

The second category is the organic aerogel route. This process typically involves the organic polycondensation of monomers such as resorcinol and formaldehyde, catalyzed in an aqueous medium to form a cross-linked organic gel (commonly referred to as an RF gel). The gel network is constructed primarily through C–C bond formation. Subsequent steps include drying, followed by carbonization under an inert atmosphere. The resulting material, known as a carbon aerogel, exhibits an exceptionally high specific surface area, which can reach values as high as 2500 m^2^/g, along with a well-developed hierarchical pore structure. Rojas-Cervantes et al.^28^ developed zirconium-loaded carbon gels through polymerization of zirconium propoxide with resorcinol and formaldehyde. The synthesis involved dropwise addition of zirconium propoxide to a solution containing resorcinol, formaldehyde and water. After 30 min of stirring and another 30 min for gelation in a sealed glass bottle placed in a silicone oil bath, the resulting hydrogel was cured at 85 °C for 7 days. The material was then crushed, dried at 110 °C under nitrogen for 10 h, and finally carbonized at 1000 °C for 5 h under nitrogen to obtain zirconium-doped AC. During the synthesis, the carbon matrix is derived from the organic polycondensation of resorcinol and formaldehyde. Concurrently, zirconium is introduced through the hydrolysis and condensation of zirconium propoxide. These simultaneous polymerization reactions result in interpenetrating networks that ensure a highly uniform distribution of zirconium throughout the composite.

The sol–gel method offers distinct advantages for preparing metal-loaded ACs, including highly dispersed metal species with strong interfacial bonding to the carbon matrix, as well as the ability to integrate multiple functional components in a single step. However, the process is relatively complex and costly, often involving expensive precursors, multiple synthesis stages, and challenges related to gel shrinkage and low carbon yield. Compared to conventional methods like impregnation or doping, the sol–gel approach provides superior control over material structure and composition, making it particularly suitable for designing advanced adsorbents with tailored performance in demanding gas adsorption applications.

### 2.4 Composites

AC can be combined with porous materials such as metal–organic frameworks (MOFs) to integrate the distinct adsorption advantages of different material systems. Adsorptive materials prepared in this way are classified as composites. Typically, finished AC is added into the preparation process of other materials to achieve deep integration, or two finished materials are simply loaded together through heat treatment (which is less common). In such composites, the advantages of both materials can be realized.

In one study, Sharafinia et al.^29^ fabricated a composite material by incorporating UIO-66, a zirconium-based MOF, with AC. The synthesis involved mixing AC with UIO-66 nanoparticles (UIONPs) at varying mass ratios (10%/20%/30%). During the synthesis of UIO-66, AC powder is added to the precursor solution (containing metal ions), and UIO-66 is grown in situ on the surface and within the pores of AC via a solvothermal method, which can be confirmed by XPS, FTIR, and SEM (Figure 4). This results in the interweaving and chemical bonding of the two components at the molecular/nanoscale, forming a homogeneous composite material. AC serves as both a structural support and an adsorption enhancer, improving the porosity, stability, and adsorption capacity of the composite material. When evaluated using gasoline vapor containing isobutane (ISO) as a model VOC, the composite demonstrated predominantly physical adsorption through the combined pore networks of both constituents, while also exhibiting excellent desorption regeneration stability and reusability. Among the tested formulations, the composite containing 20% UIO-66 displayed superior ISO adsorption capacity compared to other loading percentages. Density functional theory (DFT) calculations revealed that the enhanced adsorption originated from electron interactions between ISO molecules and zirconium sites in the composite, where the incorporation of AC with Zr_6_O_4_(OH)_4_ clusters generated positively charged zirconium atoms that facilitated electron transfer and subsequent molecular adsorption.

**Figure 4:**
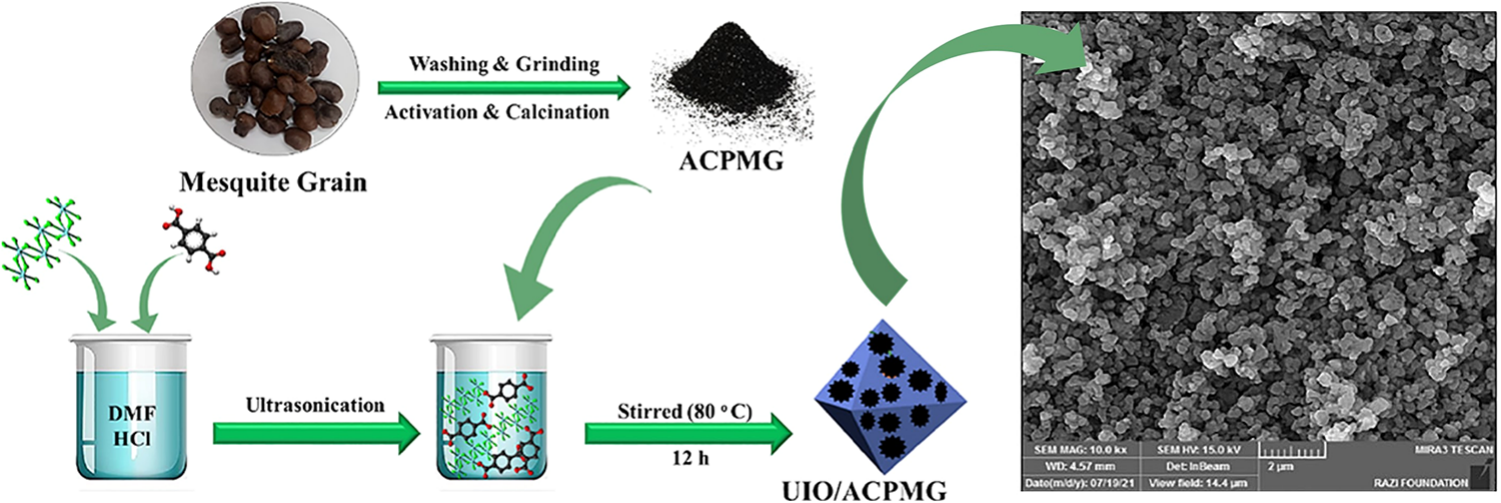
Schematic image of synthesis of AC and UIO-66 and the SEM image of the composite. (Reprinted in part with permission from ref 29, Copyright 2024, Springer Nature).

In another study, McHugh et al.^30^ synthesized a composite material by growing a copper-based MOF inside the pores of granular coal-based AC via an in situ process for ammonia adsorption. Compared with the single-component AC, the MOF–AC composite significantly enhanced the ammonia uptake capacity, increasing from 0.43 to 1.78% by weight. The composite retains the excellent physisorption capability of AC toward larger molecules such as cyclohexane, overcomes the engineering challenge of using powdered MOFs directly in filters by providing a granular form, and exhibits improved resistance to hydrolytic degradation, thereby extending the material’s usable lifetime in humid environments. The composite material exploits the coordinative interaction between the open metal sites (Cu^2+^) in the MOF framework and ammonia molecules, leading to chemisorption. EPR spectra indicate that the coupled copper paddle-wheel structure in the composite is altered or separated, generating more accessible, isolated open metal sites. This modification is likely to further promote the coordinative adsorption of ammonia molecules.

Composites can combine the strengths of both materials, offering advantages in stability, breadth of application, and enhanced adsorption capabilities. The main disadvantages include increased cost and greater process complexity.

In summary, the choice of loading method significantly impacts the final material’s properties. Impregnation offers simplicity but risks pore blockage; doping promotes uniform dispersion and pore development; sol–gel ensures intimate interfaces; and composites enable synergies with other materials. Innovative strategies, such as hybrid sol–gel with DFT-guided optimization, represent emerging universal schemes that balance dispersion, stability, and activity, outperforming traditional listing of procedures by focusing on mechanistic advantages and scalability for industrial applications.

## 3 Mechanisms of Hazardous Gas Elimination by Metal-Loaded AC

### 3.1 The Adsorption and Diffusion Process of Gaseous Adsorbate on AC

The adsorption of gases on AC proceeds through both physisorption and chemisorption mechanisms. Physisorption involves the reversible attachment of gas molecules to pore walls via van der Waals interactions. Its capacity is governed by the molecular size and boiling point of the adsorbate, as well as the specific surface area, pore volume, and pore size distribution of the carbon material.^3^ In contrast, chemisorption occurs when surface functional groups or deliberately loaded active components undergo chemical reactions—such as decomposition or conversion—with gas molecules, leading to their irreversible retention on the carbon surface in liquid or solid form. Aromatic compounds such as benzene and toluene can often be effectively removed through purely physisorptive processes. However, low boiling point, highly volatile, or recalcitrant small molecules such as dichloromethane and ethanethiol frequently require chemisorption for efficient elimination.^31^ In systems where chemisorption dominates, the activity of the loaded active components like metal species plays a decisive role in determining overall performance.

The adsorption process from the perspective of gas molecule mass transfer comprises three consecutive stages: external diffusion, internal diffusion, and surface adsorption.^1^ In the first stage, external diffusion, gas molecules migrate from the bulk phase across the boundary layer surrounding the adsorbent particles to reach their external surface. The second stage, internal diffusion, involves the transport of these molecules from the external surface into the internal pore network of the particle. As part of this stage, molecules may collide with and adhere to pore walls via internal surface adsorption. The overall rate of adsorption is determined by the slowest of these sequential steps. This rate-limiting step often shifts with operating conditions; for example, at high flow rates, external diffusion dominates, while in microporous systems, internal diffusion becomes critical.

According to fixed-bed adsorption dynamics and the mass transfer zone (MTZ) theory, a symmetrical sigmoidal breakthrough curve typically indicates physisorption dominated by internal pore diffusion, where mass transfer is the rate-limiting step, like sample ATAB-00000 in Figure 5a. In contrast, an asymmetrical curve with a prolonged tail or a secondary slope suggests the involvement of chemisorption or catalytic reactions, where surface processes become rate-determining, like sample ATAB-09005 in Figure 5b. In the work of Lee et al.,^32^ where activated carbon was impregnated with Cu/Zn/Ag/Mo-based ASZM metals and TEDA, the breakthrough curves exhibited a distinct asymmetric profile featuring an extended plateau and a second rising slope. They further constructed a reaction–convection–diffusion model that fit the experimental data well. As shown in their Figure 5, the model successfully captured these features: the extended plateau corresponded to TEDA-mediated chemisorption capacity, while the secondary slope reflected Cu/Zn-catalyzed hydrolysis with time-dependent deactivation.

**Figure 5:**
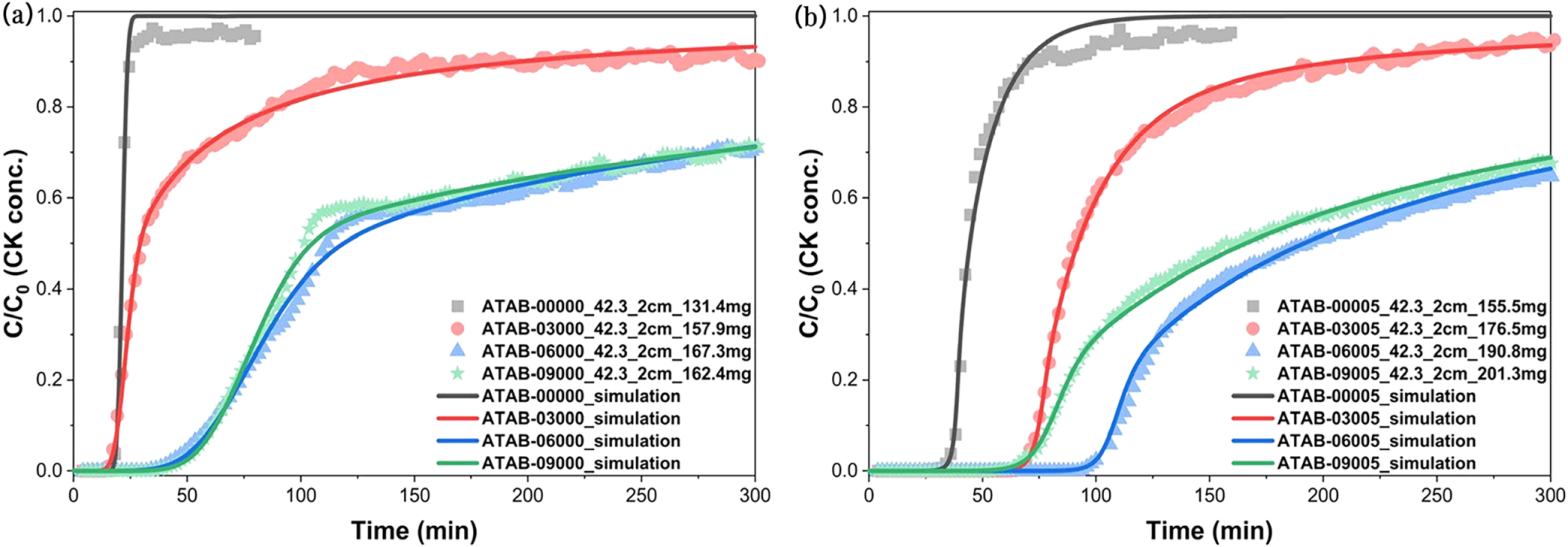
Fitted data based on the proposed reaction of CK with (a) ASZM solution; (b) ASZM-TEDA-impregnated activated carbon beads. (Reprinted with permission from ref 32, Copyright 2023, Elsevier).

When trace gas molecules arrive at and attach to the solid adsorbent surface, their motion shifts from three-dimensional free movement in space to confined two-dimensional diffusion along the surface, leading to a reduction in their degrees of freedom. On AC containing chemisorption active sites—such as metal species or surface functional groups—and under temperature conditions that enable chemical reactions, gas molecules preferentially adsorb at these active sites. In the absence of such sites or suitable conditions, only physisorption takes place. Once all active sites are saturated, additional incoming molecules are retained through physisorption mediated by van der Waals interactions with the solid surface. This sequential filling explains breakthrough curve shapes in fixed-bed adsorption, where initial chemisorption yields sharp saturation followed by gradual physisorption tailing.

The physisorption capacity of AC is fundamentally governed by its pore structure, with the microporous network playing a particularly critical role. During the internal diffusion stage, gas molecules progressively travel through macropores, then mesopores, before finally accessing the micropores. Typical ACs exhibit micropore volumes ranging from 0.25 to 0.9 cm^3^/g, with BET surface areas generally between 500 and 1500 m^2^/g, though exceptionally porous specimens may reach 3500–5000 m^2^/g. Micropores, defined as pores narrower than 1 nm, account for most of the total surface area and serve as the primary sites responsible for physisorption. In contrast, mesopores and macropores function mainly as transport channels: mesopores facilitate molecular access to the microporous regions, while macropores serve as major diffusion pathways. These larger pores also host most of the precipitated catalysts within their void spaces.^33^ During the adsorption process, micropores are filled preferentially. Once the micropores approach saturation, an adsorption film begins to form on the walls of the mesopores. Within the remaining free space of these mesopores, organic vapors may develop menisci that induce capillary condensation. It is noteworthy that even under saturated conditions, the macropore volumes remain largely unfilled by adsorbate.^34^ This hierarchical pore utilization has practical implications: in multicomponent gases, larger molecules may be excluded from micropores, leading to selectivity but also potential competitive disadvantages in humid environments.

### 3.2 Factors Influencing the Gas Adsorption Performance of AC

The gas adsorption capacity of AC depends on several key factors. These include the structural characteristics of the adsorbent, specifically its specific surface area, pore size distribution, surface functional groups, and surface charge. Additional determining factors are the catalytic activity of any loaded metal species, the physical and chemical properties of the target gas molecules, such as their size and polarity, and operational parameters including temperature, humidity, gas flow velocity, and adsorbate concentration. These factors are highly interdependent; for instance, metal loading can simultaneously enhance chemisorption via active sites while potentially compromising physisorption through pore blockage, illustrating inherent design trade-offs that must be optimized for specific applications.

#### 3.2.1 Impact of Structural Characteristics of the Adsorbent

Micropores account for over 90% of the total surface area in AC and play a dominant role in adsorption processes.^35^ In most adsorbent systems, high specific surface area, well-developed microporosity, and large micropore volume generally correspond to superior adsorption capacity.^34^ This relationship is particularly evident in the physical adsorption of large-molecule gases, where parameters such as micropore volume and surface area show strong positive correlations with adsorption performance. Yu et al.^36^ used nitrogen adsorption analysis to characterize the pore structure of differently treated ACs and examined how acetone adsorption capacity relates to key structural parameters. These included total surface area, micropore surface area, total pore volume, and micropore volume. Although all four parameters displayed linear correlations with acetone uptake, as illustrated in Figure 6, micropore surface area and micropore volume yielded higher linear regression coefficients than their total counterparts. This result further confirms that micropores serve as the primary sites determining acetone adsorption on AC.

**Figure 6:**
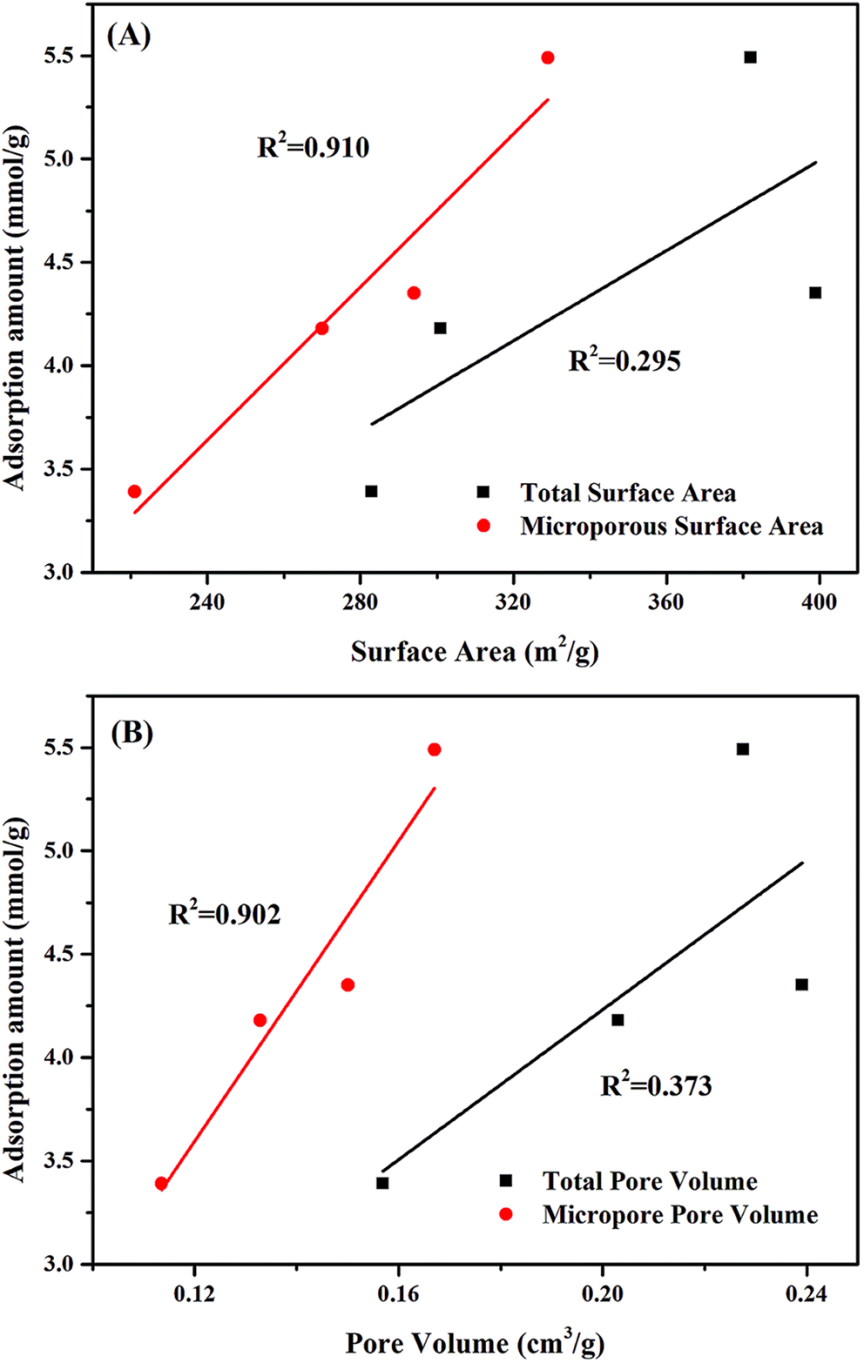
Relationship between acetone adsorption capacity and the structural properties of AC. (Reprinted with permission from ref 36, Copyright 2018, RSC Publishing).

In physisorption systems, effective adsorption requires matching pore dimensions to the molecular size of target adsorbates. While mesopores and macropores accommodate larger gas molecules, micropores demonstrate a higher affinity for smaller species.^37^ Consequently, the adsorption capacity for bulky molecules is primarily determined by the available mesopore volume. The interaction between adsorbate dimensions and pore size gives rise to four characteristic adsorption regimes:^38^ When adsorbate molecules are substantially smaller than the pore width, weak adsorption interactions result in low uptake at dilute concentrations and facile desorption. With molecular dimensions slightly smaller than the pore diameter, capillary condensation occurs, yielding significantly enhanced adsorption capacity. When molecular size closely matches the pore dimensions, a molecular trapping mechanism enables highly efficient adsorption even at ultratrace concentrations. However, adsorption is completely prevented when adsorbate molecules exceed the pore size due to molecular sieving effects. These phenomena underscore the critical importance of pore size selection relative to target molecules. Research indicates that optimal adsorption efficiency is achieved when the ratio of pore diameter to molecular diameter falls within 1.7–3.0.^5^ For regenerable systems undergoing multiple operation cycles, higher ratios of 3.0–6.0 or greater are generally necessary to maintain adequate molecular transport during repeated adsorption–desorption processes.

The loading of metal species onto AC supports directly influences the structural characteristics of the material, which in turn governs its adsorption performance toward target gases. An optimal metal content enhances adsorption capacity, whereas excessive loading typically induces pore blockage and surface area reduction, ultimately leading to diminished adsorption efficiency.

Jiang et al.^39^ investigated CuCl_2_-loaded activated carbon for NH_3_ adsorption and found that the breakthrough capacity followed the order: 5% Cu > 3% Cu > 7% Cu, indicating that increasing Cu content beyond an optimal point reduces adsorption efficiency. Structural characterization via BET, XRD, and SEM revealed that higher Cu loadings led to CuCl_2_ aggregation and pore blocking, which decreased specific surface area and pore volume, ultimately diminishing NH_3_ uptake. The adsorption curves as a function of metal content are shown in Figure 7. Similarly, Yang et al.^40^ observed that increasing the loading of Cu–Fe mixed oxides on biomass-derived char reduced both pore volume and surface area, which correlated with a decrease in Hg^0^ removal efficiency, further confirming that excessive metal loading compromises pore structure and adsorption performance.

**Figure 7:**
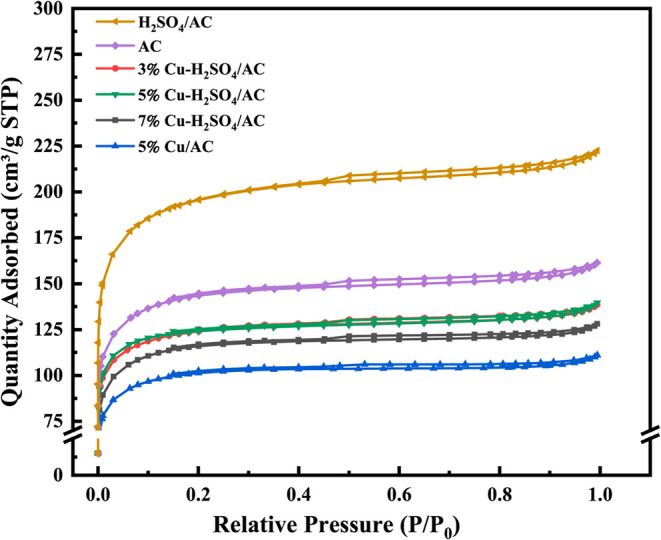
N_2_ adsorption–desorption isotherms of different samples. (Reprinted with permission from ref 39, Copyright 2025, Elsevier).

#### 3.2.2 Surface Redox Functional Groups and Acid–Base Sites

The active sites on AC adsorbents originate from defect sites in the carbon structure, specifically unsaturated carbon atoms located at the edges of graphitic basal planes.^41^ During preparation, these reactive carbon atoms bond with various heteroatoms such as oxygen, hydrogen, sulfur, nitrogen, halogens, and metal ions, leading to the formation of diverse surface functional groups. Common acidic functional groups include carboxyl, phenolic hydroxyl, and lactone groups, while typical basic groups comprise pyridinic, pyridonic, and pyrrolic structures. These acidic and basic sites preferentially interact with basic and acidic target molecules, respectively. Certain oxygen-containing functional groups enhance both the surface acidity and polarity of AC, facilitating hydrogen-bond-mediated adsorption of polar VOCs including methanol, ethanol, and acetone.^42^ Nitrogen-containing functionalities, typically introduced through treatments with ammonia, nitric acid, or nitrogenous compounds, increase the basicity of the adsorbent, thereby improving its adsorption capacity for acidic gases such as SO_2_, CO, and CO_2_.

The metal loading process modifies the surface chemical groups of AC, thereby altering its surface chemistry and adsorption properties.^43^ For instance, Lahijani et al.^44^ impregnated walnut shell–derived biochar with Mg, Al, Fe, Ni, Ca, or Na and evaluated its CO_2_ adsorption performance. The incorporation of metals introduced basic surface sites, as evidenced by XRD analysis and an alkaline leachate pH of 11.3 for the Mg-loaded sample. These basic sites promoted CO_2_ adsorption through chemisorption, likely via surface carbonate formation. Compared to pristine biochar (69.1 mg/g), the Mg-loaded variant achieved a higher CO_2_ uptake of 80.0 mg/g at 30 °C. As illustrated in Figure 8, the introduction of alkaline metal sites into the biochar framework enhanced its affinity toward acidic CO_2_ molecules.

**Figure 8:**
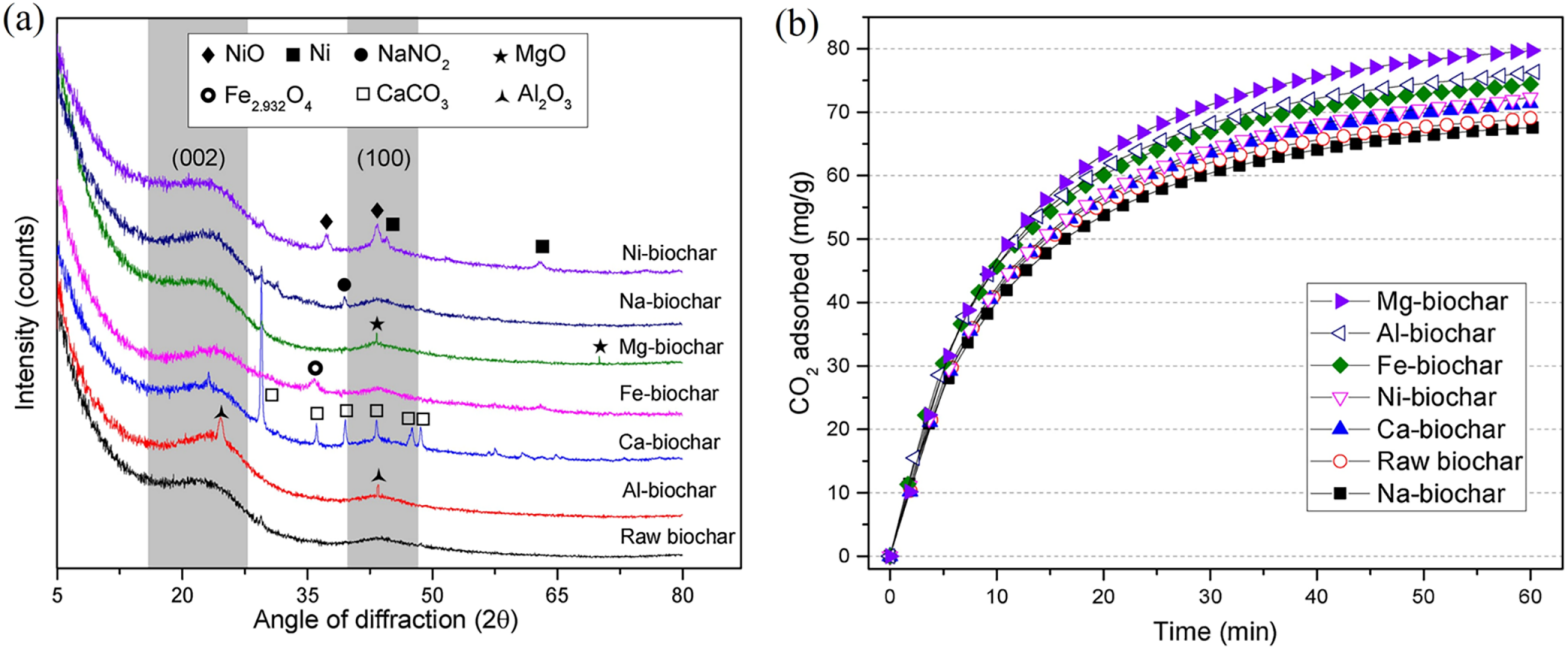
(a) XRD patterns of raw and 5 wt % metal loaded biochars and (b) CO_2_ adsorption capacity of 5 wt % metal loaded biochars at 30 °C. (Reprinted with permission from ref 45, Copyright 2018, Elsevier).

During adsorption reactions, the metal species sometimes work in tandem with the carbon framework and functional groups, exhibiting synergistic effects. A representative example can be found in a study by Wang et al.^26^ on Cr­(VI)-free spherical AC for ClCN removal. TPD/TPR/TPO-MS analyses demonstrated that doping with trace Cu­(II) or Ni­(II) ions significantly increased the density of both acid-oxidative and base-reductive sites on the carbon surface. Figure 9a is one of them. Correspondingly, the metal-doped AC achieved a superior protective time compared to its metal-free counterpart, indicating a significantly enhanced adsorption capacity for ClCN. Based on literature and experimental data, it is suggested that trace metals induce catalytic reactions in adsorption. In this process, the acid-oxidative sites on AC and metal ions interact with the negatively charged N atom of ClCN, while the base-reductive sites like superoxide anions O_2_^^–^^ on AC attract the positively charged C atom, cooperatively activating the Cl–C bond for hydrolysis (seen in Figure 9b).

**Figure 9:**
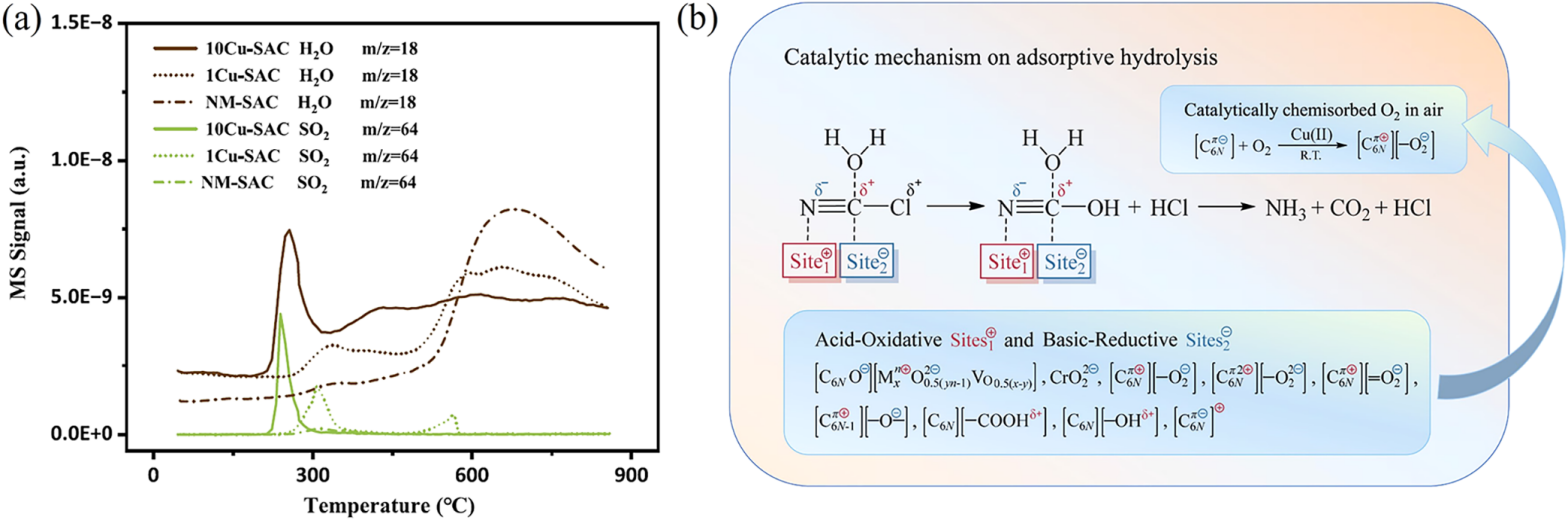
(a) MS-H_2_O and MS-SO_2_ signals in H_2_-TPR spectra for different samples and (b) catalytic mechanism on adsorptive hydrolysis of ClCN on AC. (Reprinted with permission from ref 26, Copyright 2026, Elsevier).

#### 3.2.3 Polarity

Following metal deposition, the surface of AC generally exhibits increased polarity. This enhanced polarity improves the material’s affinity for polar gas molecules, consistent with the compatibility principle in adsorption processes. When targeting nonpolar adsorbates such as gasoline vapors or larger VOC molecules, the surface polarity can be strategically reduced through thermal annealing at elevated temperatures^45^ or alkaline treatment.^46^ These processes diminish polar surface sites while developing more nonpolar characteristics, thereby optimizing the adsorbent’s affinity for specific nonpolar target compounds. Figure 10 shows the interactions between biochar and organic contaminants (polar and nonpolar). Wang et al.^47^ find that introducing Cu into the composite of MOF-199 and powdered AC (MOF-199@PAC) can produce lots of polar functional groups on the surface, which can improve better adsorption of polar VOCs like methyl ethyl ketone (MEK). For nonpolar VOCs (such as benzene), MOF-199@PAC has to rely on the nonpolar adsorption sites such as hydrophobicity and π–π stacking effects to enhance adsorption. In this study, they conclude the adsorption kinetic model for polar VOCs and nonpolar. Kinetic analysis of the adsorption process using the Boyd film-diffusion (BFD) model and intraparticle diffusion (IPD) model revealed that BFD governed the rate-limiting step for MEK adsorption on MOF-199@PAC, whereas benzene adsorption was primarily controlled by IPD. These findings provide critical insights into the mass transfer mechanisms governing the adsorption of VOCs with different polarities on the composite material.

**Figure 10:**
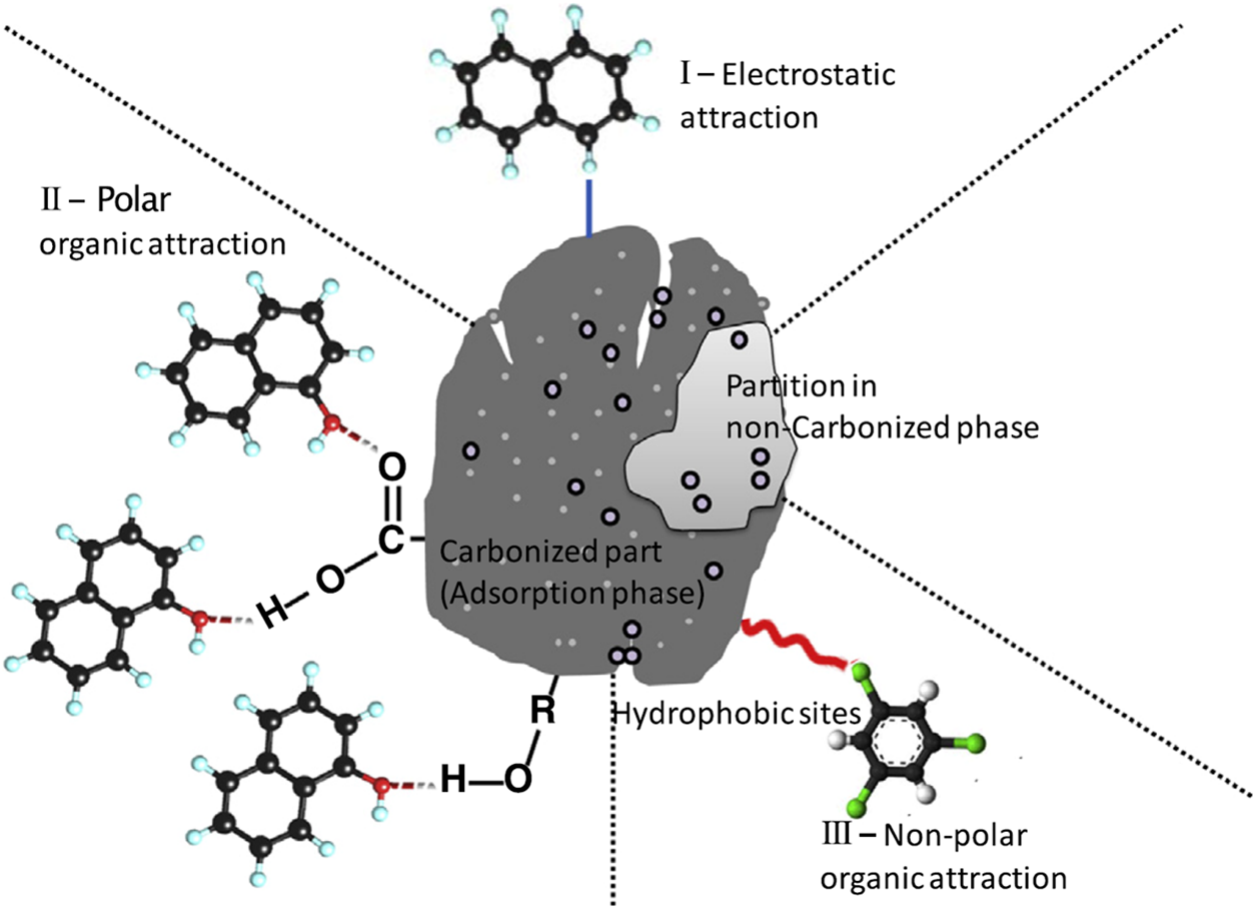
Postulated mechanisms of the interactions of biochar with organic contaminants. Circles on biochar particle show partition or adsorption. I—electrostatic interaction between biochar and organic contaminant, II—electrostatic attraction between biochar and polar organic contaminant, and III—electrostatic attraction between biochar and nonpolar organic contaminant. (Reprinted with permission from ref 49, Copyright 2014, Elsevier).

#### 3.2.4 Temperature and Humidity

Operational conditions modulate adsorption dynamics. Temperature is a critical parameter influencing both chemisorption and physisorption by altering the nature of adsorption forces. At low temperatures, chemisorption rates are minimal due to insufficient molecular activation energy, resulting in predominantly physisorption. As temperature increases, physically adsorbed gas molecules (bound by van der Waals forces) desorb, reducing physisorption capacity.^48^ Concurrently, elevated temperatures activate gaseous molecules, enhancing chemisorption. However, excessive temperatures may degrade surface functional groups or induce excessive thermal motion of adsorbates, thereby impairing chemisorption activity by destabilizing chemical bond formation. From a kinetic perspective, higher temperatures generally accelerate molecular thermal motion, increasing collision frequency between adsorbates and adsorbent surfaces to promote adsorption rates. Nevertheless, this does not necessarily translate to higher adsorption capacity, which remains constrained by adsorption equilibrium.^49^

Tang et al.^50^ synthesized a series of Cu–Zr_*x*_/Cl-BC catalysts with the application of biochar which was activated by NH_4_Cl. They found that when the loading amount of CuO-ZrO_2_ increased to 10%, the Cu–Zr_10_/Cl-BC catalyst achieved an optimal mercury removal efficiency of 98.87% at 120 °C. Furthermore, within the temperature range of 60–270 °C, the Hg^0^ removal efficiency initially increased and then decreased with rising temperature in Figure 11. This is because Hg^0^ removal primarily depends on the chemisorption (oxidation). When the temperature reached 120 °C, the Cu–Zr_*x*_/Cl-BC catalysts quickly reached chemisorption saturation, and would not adsorb more Hg^0^ even if the temperature rises. According to Zhao et al.,^43^ the mercury adsorption capacity of unmodified biochar decreased progressively with increasing temperature. In contrast, Fe/BC, FeCu/BC, and FeMn/BC exhibited an initial increase followed by a decline, with optimal adsorption occurring at 200 °C. Excessively high temperatures damaged the porous structure and deactivated adsorption sites, thereby reducing both physisorption and chemisorption. Several active sites were identified for Hg^0^ oxidative adsorption, including C=O, COOH, metal oxides and ions, lattice oxygen, chemisorbed oxygen, and Cl^–^. Among these, Fe_2_O_3_ and CuO or CuFe_2_O_4_ demonstrated a notable synergistic effect.

**Figure 11:**
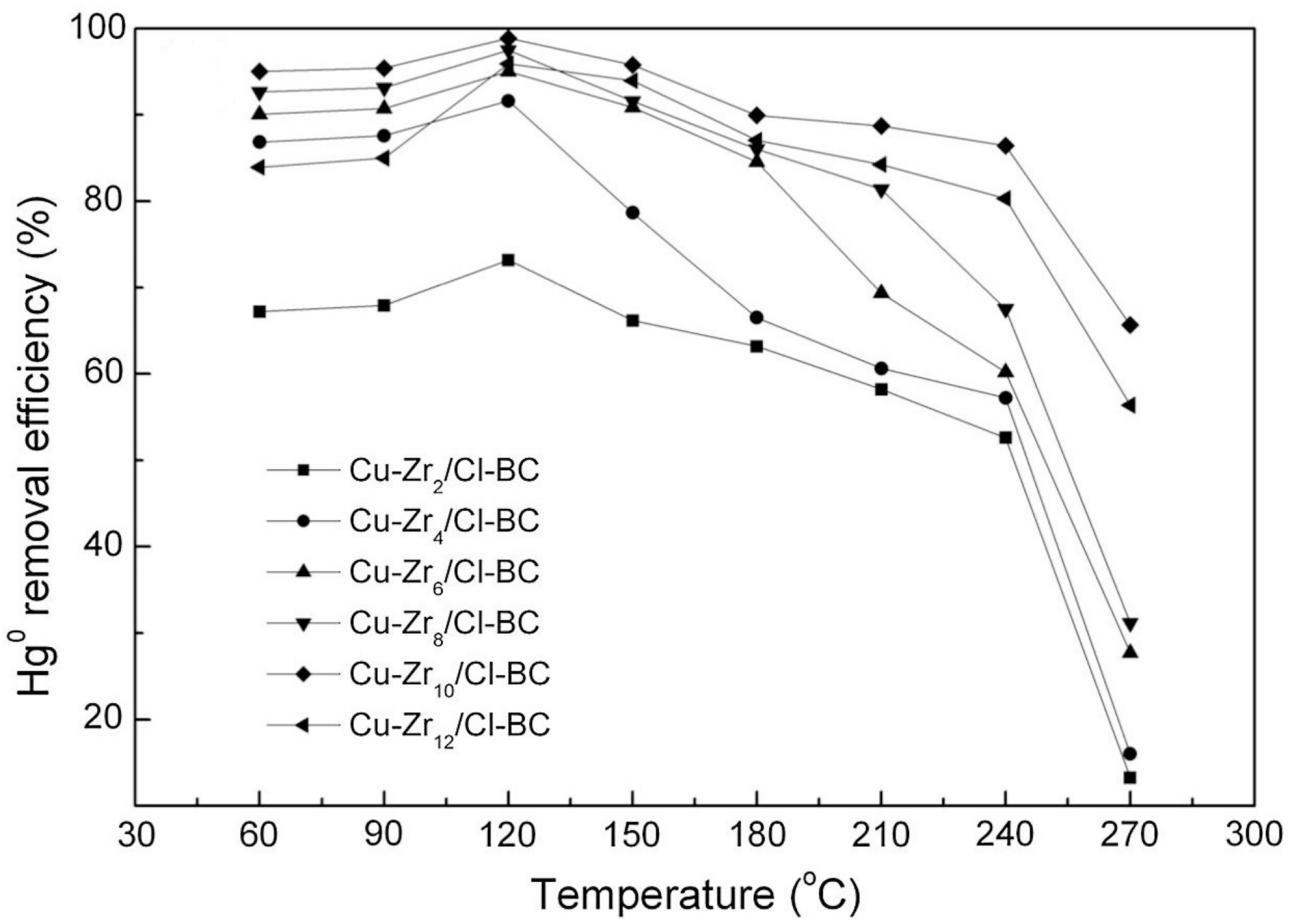
Temperature-dependent Hg^0^ removal efficiency over Cu–Zr_*x*_/Cl-BC catalysts with varying metal loading. (Reprinted with permission from ref 52, Copyright 2018, Elsevier).

Humidity primarily influences the activity of metal–supported catalysts. In chemical adsorption reactions requiring water molecule participation (e.g., hydrolytic or oxidative processes), increased humidity within an optimal range may enhance adsorption activity by facilitating reactant activation.^40^ Conversely, when competitive adsorption^51^ occurs between water and target adsorbate molecules, elevated humidity can induce catalyst aging/deactivation. Cr­(VI) which participated in the chemisorption was gradually reduced to Cr­(III) and became inactive with time in the aging experiments. In ASC whetelrite carbon used for adsorption of CWAs like cyanogen chloride, stabilizer (antiaging agent) often applied along with metal catalysts. Stabilizer may complex with metal, avoiding them reacting with oxygen and water especially when carbon is stored in a wet environment. Brown et al.^52^ found that moisture was generally detrimental to HCN uptake on impregnated AC. But when Cu (II), Cr (VI), and NaOH existed together, HCN adsorption capacity in 80% RH (5290 μmol) was far more than in dry condition (<1% RH, 3550 μmol). That is because Cu­(II) catalyzed and accelerated a chain reaction involving H_2_O to remove HCN.

Laskar et al.^53^ investigated the influence of relative humidity (RH) on the adsorption performance of AC for VOCs. The study revealed that polar VOCs exhibited greater humidity sensitivity, with significantly reduced adsorption capacities under high humidity conditions (demonstrating 16.9 and 10.7% decreases in breakthrough time for 2-propanol and acetone, respectively). In contrast, nonpolar VOCs (including toluene, n-butanol, and 1,2,4-trimethylbenzene) showed less humidity dependence, with only 0–9.6% reduction in breakthrough times, which was descripted in Figure 12.

**Figure 12:**
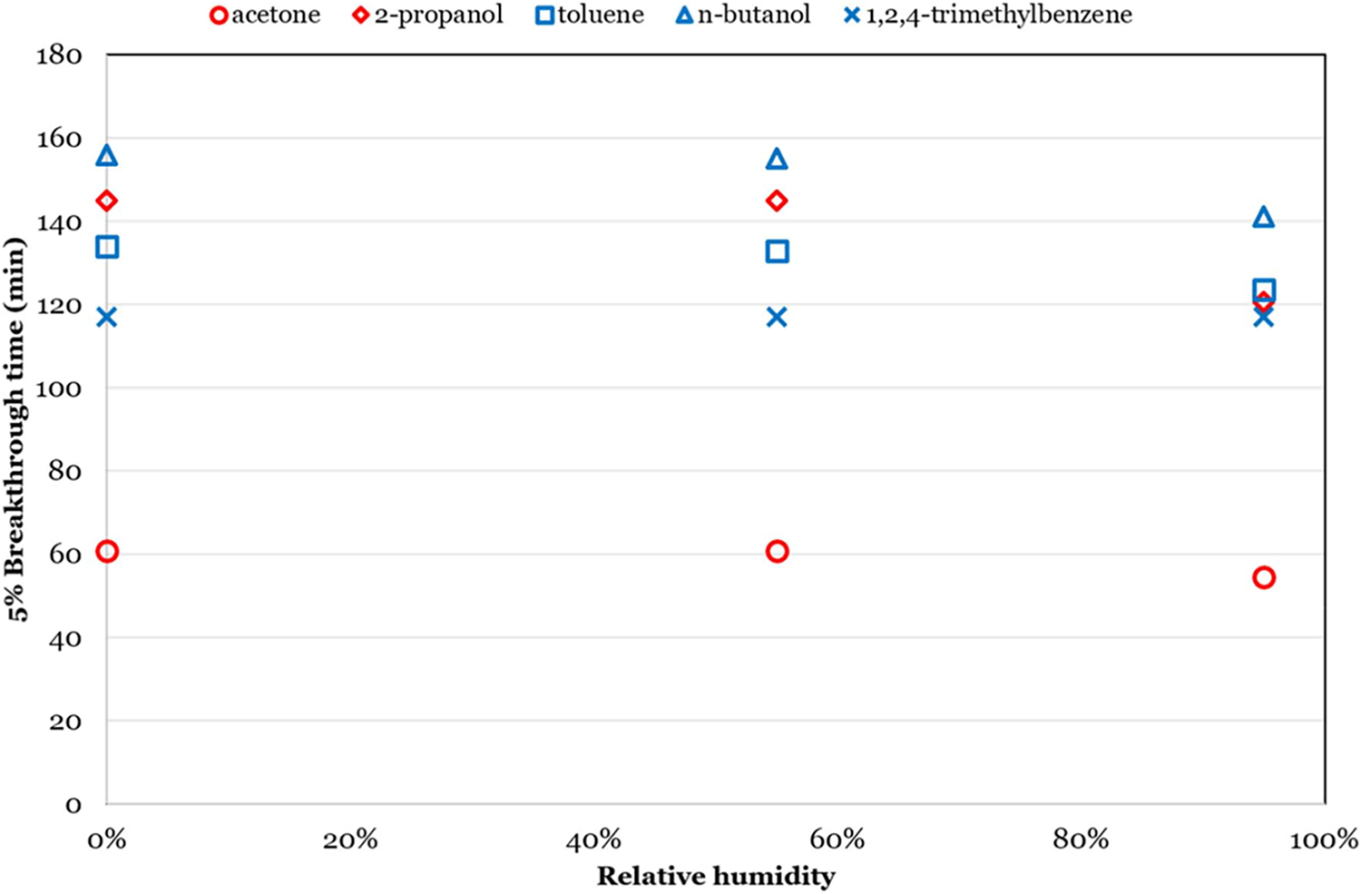
Effect of humidity (0–95% RH) on the competitive adsorption and breakthrough times of polar­(acetone, 2-propanol) and nonpolar (toluene, *n*-butanol, and 1,2,4-TMB) VOCs on AC at 298 K. (Reprinted with permission from ref 53, Copyright 2019, American Chemical Society).

#### 3.2.5 Dispersion of Metal Additives

As the metal-loaded carbon is usually used in chemisorption of gases difficult to be eliminated, the reactivity of metal additives is one of the most critical contributory factors to adsorption ability.

The method of loading metal onto AC significantly influences the binding mode between the catalyst and carbon matrix, thereby affecting both the loading efficiency and dispersion uniformity—determining the reactivity of the metal.^24^ Smaller catalyst particles with higher dispersion and greater exposed surface area accelerate catalytic reaction rates, leading to extended breakthrough times under equivalent gas flow conditions and enhanced chemisorption activity. Optimized loading methods can achieve more uniform and deeper catalyst dispersion. Alternative methods like doping, sol–gel, and chemical vapor deposition inherently yield superior metal particle dispersion owing to their unique preparation mechanisms. Xu et al. doped FeCl_3_ into hickory chips to yield AC with Fe oxyhydroxide for CO_2_ capture. It was found that postpyrolysis ball milling was an effective step to improve CO_2_ capture by downsizing the carbon particles, introducing their abundant surface defects and bettering Fe dispersion. Consequently, the adsorption equilibrium time was shortened from >1200 to 150–600 min while maintaining high sorption capacities (>150 mg/g). Figure 13 illustrates the evolution of Fe speciation, adsorption mechanism and uptake rate. It should be noted that, under low-Fe conditions, physisorption proceeds rapidly; however, its capacity is only about 1/3 of that achieved via chemisorption in Fe-rich composites.

**Figure 13:**
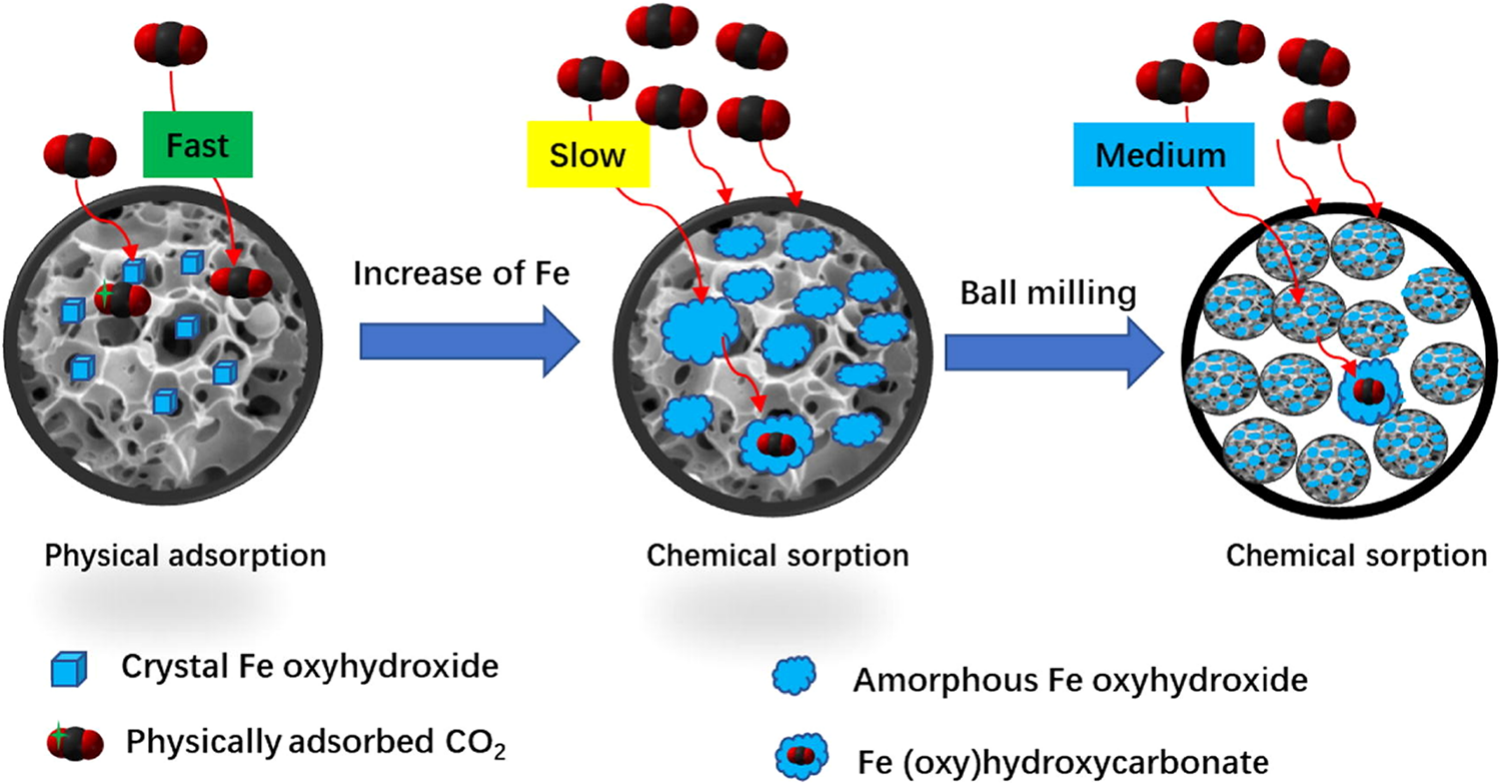
Effect of ball milling on metal speciation/dispersion, adsorption mechanism and uptake rate. (Reprinted with permission from ref 56, Copyright 2020, Elsevier).

Notably, the total metal dosage does not directly correlate with effective loading capacity. For instance, during impregnation, increased metal addition may fail to improve adsorption performance due to either surface saturation or pore blockage from poor metal dispersion.^54^ Furthermore, loaded metals may exist in multiple valence states, potentially appearing as metal oxides/sulfides, or form stronger interactions like metal–carbon bonds with the carbon matrix and surface acid–base sites.^55^ These configurations can enhance electron transfer capacity and synergistic coupling with the carbon support so as to improve overall adsorption performance.

The dispersion uniformity of metal species is also significantly influenced by preparation conditions, particularly the carbonization temperature. Qin et al.^4^ found that the walnut shell based carbon (WSC) carbonized at 700 °C had more surface defects and a larger specific surface area than at other temperature. After being impregnated with Fe, it was the WSC carbonized at 700 °C that got highly dispersed Fe species particles and an advantage in the removal of SO_2_, NO_*x*_, and Hg^0^.

### 3.3 Chemisorption Mechanisms of AC Loaded Metals

The bonding behavior and catalytic properties of d-block metal ionsare primarily governed by their partially filled or energetically accessible (*n*–1) d orbitals, along with the vacant ns and np orbitals of the valence shell. This set of orbitals enables hybridization and the formation of molecular orbitals during bonding with ligands or absorbates. The d-orbitals exhibit a unique electronic configuration, enabling simultaneous formation of both σ- and π-bonds—a key factor contributing to the catalytic properties of transition metals and their complexes.^56^ The mechanistic principles governing transition metal-based gas adsorption reactions, whether through supported catalysts or direct participation, can be systematically categorized as follows: (1) Chemisorption and coordination effects. The d-orbitals of transition metals undergo orbital overlap with adsorbate molecules (e.g., HCN, CO), forming coordination or covalent bonds that enable highly selective and robust chemisorption. (2) Electron transfer and activation capability. The partially filled d-orbitals in transition metals’ outermost shells function as electron donors or acceptors, modulating adsorbate electron density distribution through electron donation/withdrawal. This electronic perturbation reduces reaction activation barriers and promotes molecular activation. (3) Multivalent redox and synergistic effects. Transition metals exhibit variable oxidation states (e.g., Fe^2+^/Fe^3+^, Cu^+^/Cu^2+^) that enable reversible interconversion, working synergistically with support materials to establish efficient electron transfer pathways for redox processes.

During the preparation of metal-loaded AC, metal particles may form coordination complexes with surface functional groups such as alkyl (−R), carboxyl (−COOH), amide (−CONH_2_), hydroxyl (−OH), carbonyl (>C=O), phenolic (−PhOH), and alkyne (−C≡C−) groups, or attached to the surface of carbon forming nanoparticles of metal oxides, carbides, sulfides, etc. These fixed metal nanoparticles within the pores can modify the carbon surface and act as nucleophilic or electrophilic sites effectively increasing adsorption reactivity. The introduction of metal ions onto the carbon surface or into pore structures can also induce pore expansion through reactions with surrounding carbon atoms.^57^ Following intraparticle diffusion, the adsorbed gas molecules interact with metal particles on the AC surface, where the metals may either catalyze the decomposition of gases by oxygen/water or directly participate in decomposition reactions, ultimately converting gaseous pollutants into solid or liquid products fixed on the carbon surface,^58^ as descripted in Figure 14. Importantly, the AC support not only serves as a gas transport channel and metal carrier but also facilitates electron transfer through its sp^2^/sp^3^ hybridized carbon network, creating synergistic effects with metal active centers^24^ that significantly enhance the capture and conversion efficiency of adsorbate molecules.

**Figure 14:**
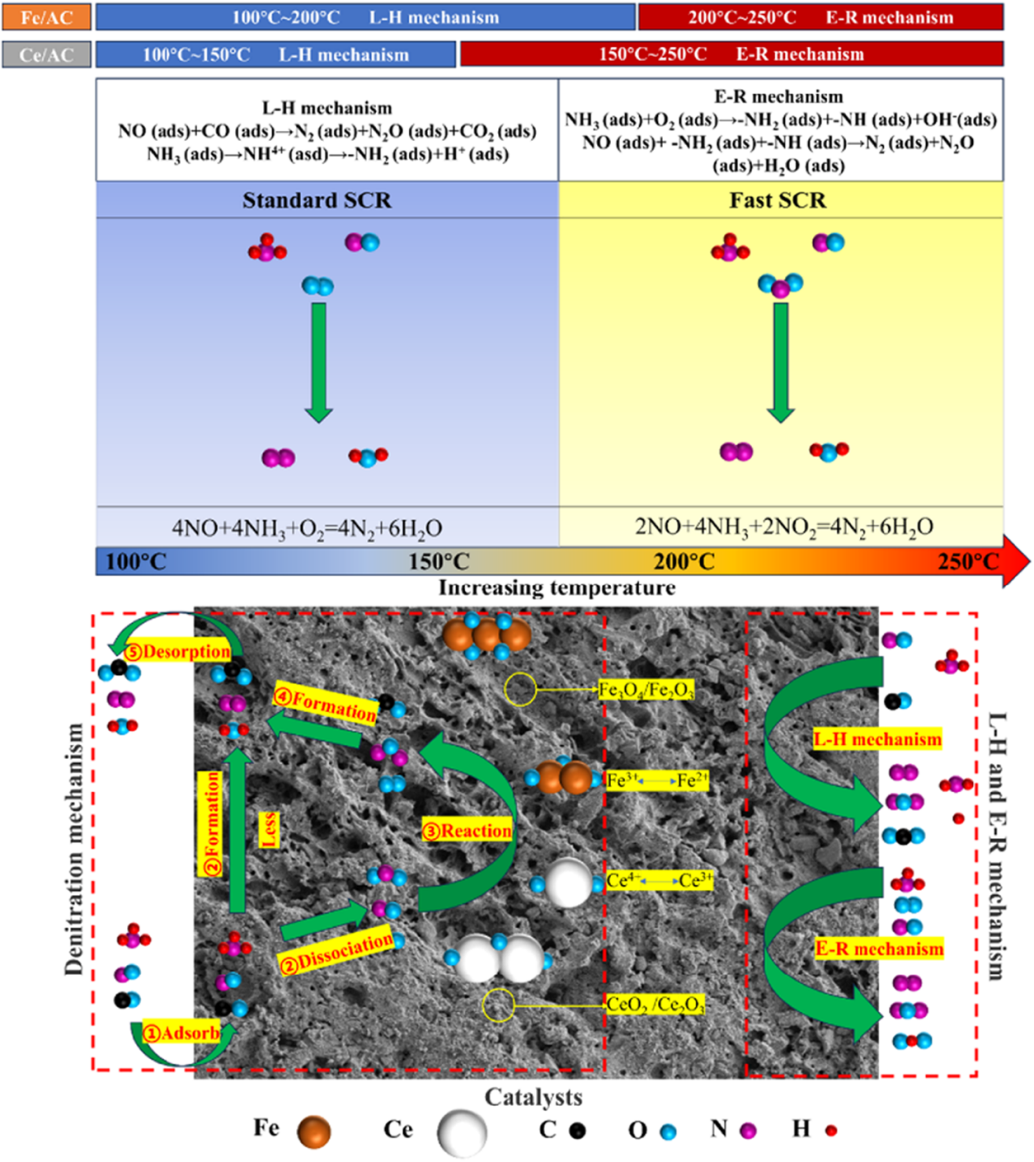
Mechanism of NO removal by NH_3_ + CO coupling at low temperature. (Reprinted with permission from ref 59, Copyright 2025, Elsevier).

## 4 Application of Hazardous Gas Adsorption on AC Loaded Metals

The removal of hazardous gases by metal-loaded AC can be classified into several categories based on the target pollutants: desulfurization (removal of sulfur compounds), denitrification (removal of nitrogen oxides), carbon monoxide/carbon dioxide removal, and elimination of small molecular toxic agents. Table [Table tblA1] lists some of the applications of metal-loaded AC for the elimination of diverse hazardous gases.

**A1 tblA1:** Applications of Metal-Loaded AC for the Removal of Diverse Hazardous Gases

Materials	Metal	Loading method	Ads gas	Properties of AC	Removal effects and conditions	ref
Manganese acetate and tetrabutyltitanate	Mn/Ti	Sol–gel	VOC	*S*_BET_: 739.7–829.8 m^2^/g	With a toluene removal efficiency of 86% and a CO_2_ yield of 116 ppm, 0.1%Mn/40%TiO_2_/AC was identified as the most active and stable catalyst for toluene degradation across different TiO_2_ loadings. This formulation also exhibited a superior capacity for ozone decomposition.	^93^
Carboxymethylcellulose sodium and iron alum	Fe/Na	Sol–gel	VOC	*A*_micro_: 3–950 m^2^/g, pore volume: 0.03–0.52 cm^3^/g	c900-CFeA0.05 and c900-CFeA0.15 achieved >90% removal for most VOCs. c700-CFeA1.0 (20.8 wt % Fe) showed lower removal ratios (25–83%), attributed to pore blockage by excess Fe particles.	^94^
Unknown	Cu	Impregnation and calcination	VOC	*S*_BET_: 483.8–609.3 m^2^/g, pore volume: 0.32–0.37 cm^3^/g	The multifactor orthogonal experiment showed that the influence degree of various factors for benzene elimination was: reaction space velocity ≫ CuO loading > energy density > inlet benzene concentration ≫ reaction humidity. The highest benzene removal efficiency achieved 96.5%.	^95^
Unknown commercial AC	Mg/Zn/Cu/Zr	Impregnation and calcination	VOC	*S*_BET_: 769–847 m^2^/g, pore volume: 0.36–0.41 cm^3^/g	The deposition of metal oxide nanoparticles significantly enhanced the adsorption capacity for acetone and methanol compared to pure AC. Among the composites, AC/ZnO demonstrated the most superior performance, with capacities reaching 415 mg/g for acetone and 481 mg/g for methanol at 25 °C.	^85^
Unknown granular AC (GAC)	Zn	Impregnation	VOC	*S*_BET_: 1042 m^2^/g, pore volume: 0.4471 cm^3^/g	The benzene removal efficiency of both processes increased with the ZnO-GAC dosage raised from 0 to 10 mg at 25 °C. Specifically, the efficiency improved from 42.07 to 70.24% for the UV/ZnO-GAC process, and from 60.9 to 89.57% for the O_3_/ZnO-GAC/UV process.	^60,96^
Mixture of coal and coconut shell	Fe/Co/Ni/V/Mn/Cu/Ce	Impregnation	SO_2_	*S*_BET_: 621–824 m^2^/g, micropore volume: 0.31–0.42 cm^3^/g	At 150 °C, V-AC showed a markedly superior SO_2_ adsorption capacity of 65 mg/g, greatly outperforming other metal-AC composites with the trend Ni-AC < Co-AC < Fe-AC.	^97^
Bituminous coal, coking coal	Cu	Doping (one-step carbonization–activation)	SO_2_	*S*_BET_: 403–520 m^2^/g, pore volume: 0.215–0.281 cm^3^/g	The desulfurization activity increased significantly with CuO loading and peaked at 6 wt %. At this optimal loading, the composite achieved a sulfur capacity of 219.2 mg/g and a breakthrough time of 26.2 h at 80 °C in flue gas, substantially outperforming the naked AC (120.1 mg/g, 14.8 h).	^98^
Bituminous coal, coking coal	Co/Ti/Mn/Fe/V/Ni/Cu	Doping (one-step carbonization–activation)	SO_2_	*S*_BET_: 356–475 m^2^/g, pore volume: 0.191–0.274 cm^3^/g	Co_2_O_3_ and Fe_2_O_3_ achieved good modification activity of 163.9 and 137.9 mg/g respectively at 80 °C in flue gas. Among the series of monometallic modified AC catalysts, the desulfurization activity decreased in the order: AC < V5/AC < Ti5/AC < Cu5/AC < Ni5/AC ≈ Mn5/AC < Fe5/AC < Co5/AC.	^99^
Coffee residue	Cu	Impregnation	H_2_S	*S*_BET_: 1051.5–1422.1 m^2^/g, pore volume: 0.417–0.655 cm^3^/g	The Cu/AC filter exhibited a high H_2_S adsorption capacity of 132.22 mg/g at 20 °C. This performance was significantly enhanced by the copper-impregnation process, which introduced functional groups—notably oxygen-containing groups such as O–H and C–O—that accelerated the adsorption rate and improved overall efficiency.	^97^
Coal coke	V	Impregnation	SO_2_	*S*_BET_: 306–700 m^2^/g, pore volume: 0.13–0.28 cm^3^/g	At temperatures near 200 °C, V_2_O_5_/AC shows greater SO_2_ removal activity from flue gases than activated cokes (AC), as V_2_O_5_ supplies lattice oxygen for SO_2_ absorption and oxidation into a VOSO_4_-like intermediate.	^100^
Corncob	Cu/Zn	Polyol	CO_2_	*S*_BET_: 1738–1881 m^2^/g, pore volume: 0.783–0.854 cm^3^/g	Cu–Zn/AC, demonstrated the highest CO_2_ capture capacity of 5.41 mmol/g compared to the parent AC (3.25 mmol/g) as well as the single metal-doped ACs, Cu/AC (4.19 mmol/g) and Zn/AC (4.38 mmol/g) at 1 bar and 25 °C due to stronger synergistic effects.	^101^
Unknown AC (Norit@ SA2, Sigma–Aldrich)	Cu/Zn	Impregnation	CO_2_	*S*_BET_: 621.90–925.07 m^2^/g, micropore volume: 0.399–0.587 cm^3^/g	The breakthrough time at 30 °C raised from 32 to 45 min when the loading amount of Cu/Zn raised from 4 to 16%. An approximate 49% increase in CO_2_ adsorption capacity was achieved by the Cu/Zn-20% sample compared to unmodified AC, in addition to the observed superiority of Cu-loaded over Zn-loaded AC.	^102^
polystyrene (PSI)- based ion-exchangeable resins	Cu	Electroplating and postoxidation	CO_2_	*S*_BET_: 1510–1590 m^2^/g, pore volume: 0.86–0.89 cm^3^/g	The electroplated metallic copper loaded carbon lowered CO_2_ adsorption capacity value than the as-received carbon, while the postoxidated one increased. It was due to the alkalinity of Cu_2_O, CuO and the acidity of CO_2_. An excessive amount of copper oxide does not accelerate the adsorption ability.	^103^
Cottonwood	Al/Fe/Mg	Impregnation	CO_2_	*S*_BET_: 184–749 m^2^/g, pore volume: 0.86–0.89 cm^3^/g	AlCW(4) sample had the highest adsorption of 71.05 mg/g at 25 °C, with FeCW(6) 65.26 mg/g and MgCW(0.1) 63.69 mg/g. An increase in the amount of metal did not necessarily lead to an increase in adsorption capacity.	^104^
Walnut shell	Mg/Al/Fe/Ni/Ca	Impregnation and heat treatment	CO_2_	*S*_BET_: 94.509–397.015 m^2^/g, pore volume: 0.054–0.198 cm^3^/g (only data of nonmetal)	The CO_2_ capture capacity of metal-modified biochar followed the order: Mg > Al > Fe > Ni > Ca > raw biochar > Na. At 25 °C, the uptake by Mg-biochar reached 82.0 mg/g, which is 15.7% higher than that of the raw biochar (72.6 mg/g).	^105^
Pinecone	Cu/Mn	Impregnation	HCHO, Hg^0^	*S*_BET_: 308.94–320.98 m^2^/g, pore volume: 0.14–0.16 cm^3^/g	Optimal removal performance for HCHO (89%) and elementary mercury (83%) was observed at 175 °C using biochar-supported Cu–Mn mixed oxides.	^106^
Walnut Shell	Fe/Cu/Mn	Coprecipitation	Hg^0^	*S*_BET_: 184.18–278.64 m^2^/g, pore volume: 0.031–0.079 cm^3^/g	The adsorption capacities of Fe/BC, FeCu/BC, and FeMn/BC all enhanced first and then weakened with temperature, with 200 °C the optimum. FeCu/BC had best adsorption capacity of 3901 ng/g at 200 °C.	^43^
Walnut Shell	Fe/Cu/Mn	Coprecipitation	Hg^0^	*S*_BET_: 9.27–288.76 m^2^/g, pore volume: 0.019–0.082 cm^3^/g	The adsorption capacities of Fe-2% Mn/BC adsorbent was the highest with Q value of 4141 ng/g at 50 °C.	^24^
Cotton straw	Mn/Fe	Impregnation	Hg^0^	*S*_BET_: 401.037–451.569 m^2^/g, pore volume: 0.2495–0.3011 cm^3^/g	An optimal Hg^0^ removal efficiency of 87.1% was achieved at 120 °C by the MnFe4% (3/10)/CSWU700 adsorbent, with chemisorbed oxygen (O_β_) playing a vital role in the oxidation process.	^49^
Wheat straw	Fe/Cu	Impregnation	Hg^0^	*S*_BET_: 191.388–286.494 m^2^/g, pore volume: 0.209–0.291 cm^3^/g	At 130 °C, the CuFe0.3/WSWU10(500) adsorbent achieved a high Hg^0^ removal efficiency of about 90.58%.	^40^
Peanut shell	Mn/Ce	Impregnation	NO	*S*_BET_: 378.962–405.823 m^2^/g, pore volume: 0.033–0.049 cm^3^/g	At 175 °C, the 6% Mn–Ce (7:3)/BC catalyst exhibited optimal activity, reaching a NO conversion of 99.2%.	^107^
Lignocellulosic/herbaceous	K/Cu/Fe/Ni	Impregnation	NO_*x*_	*S*_BET_: 372–451 m^2^/g	At temperatures exceeding 250 °C, the process transitioned from pure adsorption to NO reduction. Among the tested materials, SAC-K was uniquely selective toward facilitating NO_*x*_ reduction in the presence of O_2_.	^108^
Unknown AC	Fe/Mn/Ce/La	Impregnation	NO–CO	*S*_BET_: 28.27–158.97 m^2^/g, pore volume: 0.10–0.17 cm^3^/g	A strong synergistic interaction between Fe and Mn in the Mn-doped 10Fe/AC catalyst significantly enhanced its redox properties. This was manifested through improved FeO_*x*_ dispersion, abundant oxygen vacancies, and a higher surface-adsorbed oxygen (O^α^) content, leading to superior catalytic performance with 97.5% NO conversion and 83.3% CO removal at 240 °C.	^109^
Empty fruit bunch (EFB)	Cu	Impregnation	NO_*x*_	*S*_BET_: 606–699 m^2^/g, pore volume: 0.26–0.30 cm^3^/g	The effect of Cu impregnation on NO_*x*_ removal was temperature-dependent. At lower temperatures, it inhibited the process; however, at elevated temperatures above 150 °C, the intrinsic catalytic activity of Cu led to a notable improvement.	^110^
Coconut shell	Fe/Ce	Doping	NO–CO	*S*_BET_: 622–644 m^2^/g, pore volume: 0.307–0.323 cm^3^/g	At lower temperatures, the Fe–Ce/AC catalyst achieved and sustained a peak NO conversion of 99.9%. As the temperature exceeded 125.5 °C, the conversion efficiency began to decrease progressively.	^59^
Alginate/AC	Cu/Zn/Mo/Ag	Composite	ClCN, Sarin	*S*_BET_: 496–905 m^2^/g, pore volume: 0.25–0.42 cm^3^/g	The breakthrough time of AC0605 (with 6% Cu and 5%TEDA) against sarin enhanced from 86.8 to 124.7 min than AC with no metal. The breakthrough time against cyanogen chloride enhanced 128% from 71.8 to 92.1 min in the condition of MIL-DTL-32101 standard.	^111^
Unknown AC beads	Cu/Zn/Mo/Ag	Impregnation	ClCN	*S*_BET_: 627.13–1417.36 m^2^/g, pore volume: 0.29–0.68 cm^3^/g	The pure carbon substrate exhibited a breakthrough time of approximately 18 min at 24 °C. The incorporation of TEDA demonstrated a notable extension, with 5 and 10% loadings yielding breakthrough times of 27 and 33 min, respectively. Carbon with 6% Cu, 6% Zn, 2.4% Mo, 0.061% Ag and 5% TEDA had the best ClCN adsorption ability.	^32^
Apricot shells	Cu/Zn/Mo/Ag	Impregnation	Cl_3_CNO_2_, COCl_2_, HCN	*S*_BET_: 776–892 m^2^/g, pore volume: 0.805–0.861 cm^3^/g	Carbon impregnated with Cu/Cr/Ag and TEDA had the longest breakthrough time against COCl_2_ of 29.9 min at 24 °C, while the starting one with no metal had the best breakthrough time against Cl_3_CNO_2_ of 73.31 min and the one with Cu/Zn/Cr/Ag and 4% K_2_CO_3_ had the longest breakthrough time against HCN of 54 min.	^112,113^
Unknown commercial AC	Fe/Cu/Co/Mn/Ni	Impregnation	HCN	*S*_BET_: 1196.5–1238.2 m^2^/g, pore volume: 0.1841–0.1906 cm^3^/g	The HCN conversion efficiency increased markedly with temperature, reaching near-complete conversion (exceeding 98%) at temperatures of 250 °C or higher. Among the tested catalysts (AC-Mn, AC-Co, AC-Ni, AC-Fe, AC-Cu), AC-Cu exhibited a distinct advantage over a wide temperature range.	^114^
Coal-based carbon	Cu/Zn/Mo/Ag	Impregnation	ClCN	*S*_BET_: 687–734 m^2^/g, micropore volume: 0.288–0.305 cm^3^/g	Phosphate addition, particularly NaH_2_PO_4_, exhibited significantly inhibited aging in the Cu/Zn/Mo and TEDA-impregnated AC for ClCN removal. The protection value declined by only ∼20% postaging, in sharp contrast to a drastic 96% loss in the absence of the additive.	^115^

### 4.1 Volatile Organic Compounds (VOCs) Elimination

Lu et al.^19^ prepared Cu-, Co-, Fe-, and Ni-loaded coconut shell ACs via impregnation for VOC (toluene as representative) and NO removal. Appropriate concentrations of metal nitrate solutions were stirred at until evaporation to obtain catalysts with approximately 3 wt % metal content, followed by drying and thermal treatment under hydrogen to obtain reduced catalysts. The study found that at low temperatures (200–250 °C) in the presence of oxygen, transition metal-loaded AC could catalytically oxidize VOCs completely to CO_2_ and H_2_O. At 250 °C, Co/AC and Cu/AC showed higher activity for deep VOC oxidation. As VOC concentration increased on the AC, VOCs migrated from the surface to active sites, enhancing oxidation rates. In the presence of both VOCs and oxygen, the transition metal-loaded AC also catalyzed NO reduction to N_2_. The AC not only served as a support material to disperse active sites for NO distribution but also participated as a reductant in NO decomposition reactions.

Jafari et al.^60^ prepared nanozinc oxide impregnated granular AC (ZnO-GAC) for benzene degradation through photocatalytic ozonation. The catalytic performance of ultraviolet (UV) irradiation, ozone (O_3_) oxidation, and their synergistic coupling with ZnO-GAC was systematically evaluated for benzene degradation in contaminated air streams. Results revealed that the UV/ZnO-GAC process attained markedly superior removal efficiencies, predominantly attributed to the pronounced photocatalytic activity of the ZnO-GAC nanocomposite. When exposed to UV light of sufficient energy, photoexcitation of the catalyst generates electron–hole pairs. The highly reductive excited electrons then produce hydroxyl radicals, which, together with the positive holes, subsequently enable the degradation of pollutants (eqs 1–7). A similar mechanism was reported by Shu et al.^61^ in Figure 15, who synthesized Mn/TiO_2_/AC via a sol–gel method for the photocatalytic removal of toluene. The Mn/TiO_2_/AC catalyst not only adsorbs toluene but also efficiently decomposes O_3_ and converts it into hydroxyl radicals (•OH). This enables a quadruple synergy of adsorption, photolysis, catalytic oxidation, and O_3_ utilization, leading to a toluene degradation efficiency ofapproximately 86% and complete elimination of O_3_.

1 $$\mathrm{ZnO‐}\mathrm{GAC}+\mathrm{hv}\to \mathrm{ZnO‐}\mathrm{GAC}\left(h_{\mathrm{VB}}^{+}\right)+Zn\mathrm{O‐}GAC\left(e_{\mathrm{CB}}^{-}\right)$$

$$O_{2}+\mathrm{ZnO}‐\mathrm{GAC}\left(e_{\mathrm{CB}}^{-}\right)\to O_{2}^{-}•$$ 2

3 $$O_{2}^{-}•+H^{+}\to {\mathrm{HO}}_{2}•$$

4 $${\mathrm{HO}}_{2}•+O_{2}^{-}•\to +{{\mathrm{HO}}_{2}}^{-}+O_{2}$$

5 $$2{\mathrm{HO}}_{2}•\to O_{2}+H_{2}O_{2}$$

6 $$H_{2}O_{2}+\mathrm{hv}\to 2•\mathrm{OH}$$

7 $$H_{2}O_{2}+e_{CB}^{-}\to {\mathrm{OH}}^{-}+•\mathrm{OH}$$

**Figure 15:**
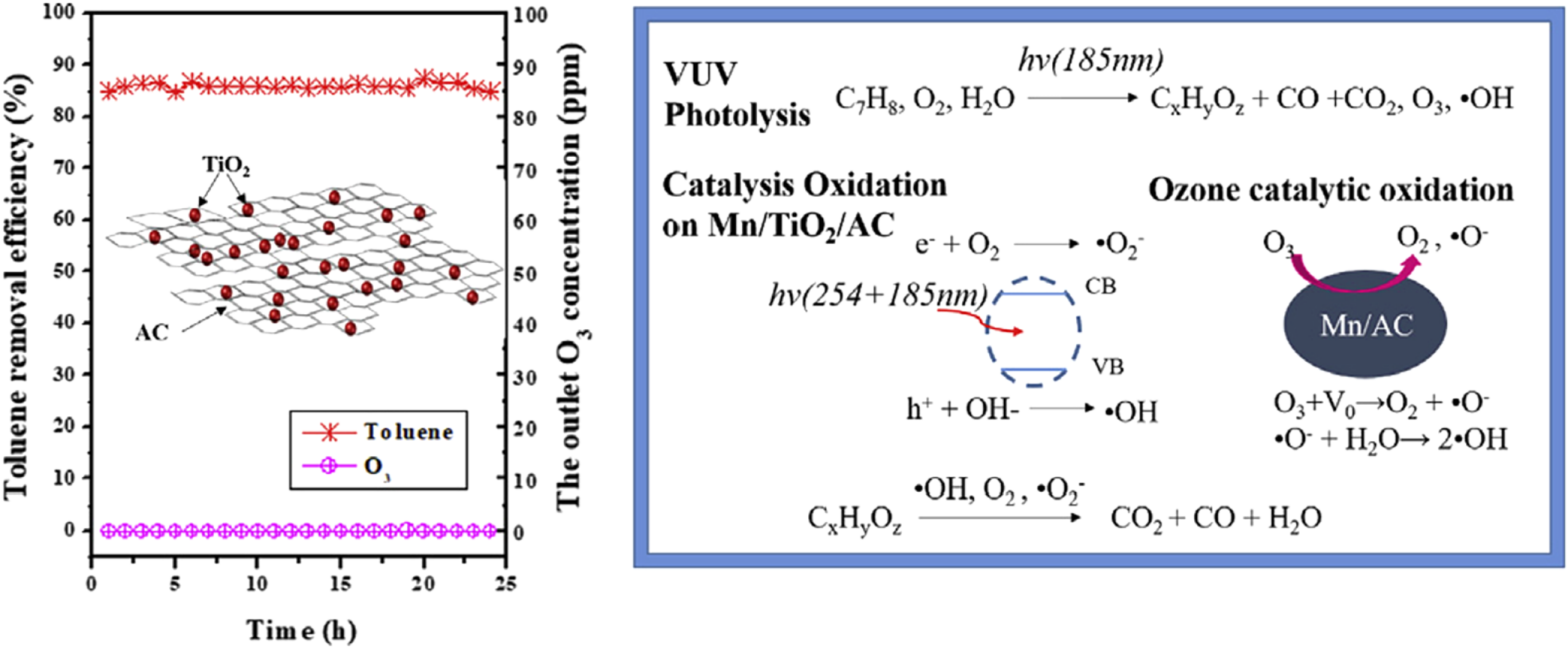
Schematic of VOC Adsorption and Photocatalytic Oxidation on Mn/TiO_2_/AC. (Reprinted with permission from ref 64, Copyright 2018, Elsevier).

### 4.2 Nitrogen Oxides (NO_*x*_) Elimination

Illán-Gómez et al.^62^ prepared coal-based AC loaded with iron (Fe, 4.7 wt %), copper (Cu, 3.0 wt %), chromium (Cr, 2.0 wt %), cobalt (Co, 3.7 wt %), and nickel (Ni, 3.5 wt %) via impregnation for catalytic NO reduction. The samples were obtained by impregnation with excess metal nitrates (10 mL/g AC), followed by nitrogen bubbling for drying and oven treatment. The study found that all five metal-loaded catalysts could promote NO reduction, significantly lowering the activation energy and enabling the reaction to occur at lower temperatures. The researchers proposed that metals with unpaired electrons (paramagnetic or ferromagnetic metals like Fe, Co, and Ni) could interact with the unpaired electrons in NO molecules to form metal-NO species, thereby facilitating NO reduction. During the reaction, metals first adsorbed NO and underwent redox reactions—the metals were oxidized while NO was reduced. Subsequently, the carbon support reduced the metal oxides back to metallic states, transferring oxygen from the metal oxides to carbon to form CO and CO_2_, while regenerating the metals for further NO adsorption. The oxygen transfer process was identified as the key step, where the reducibility of metal oxides determined the oxygen transfer rate and consequently affected the overall catalytic reaction rate.

Wen et al.^1^ summarized catalytic reduction processes of NO_*x*_ on metal-doped AC. The reaction was identified to follow the Eley–Rideal (E–R) mechanism, involving adsorbed NH_3_ and gaseous NO. Specifically, NH_3_ first adsorbs rapidly onto Lewis acid sites as coordinated NH_3_ on the MnO*_x_* surface. This coordinated NH_3_ then interacts with surface-adsorbed oxygen to form -NH_2_ species. Subsequently, the −NH_2_ intermediate reacts with gaseous NO to produce NH_2_NO, which ultimately decomposes into N_2_ and H_2_O (eq 8).^63,64^ Another reaction pathway, known as the Langmuir–Hinshelwood (L–H) mechanism, involves the interaction between adsorbed NH_3_ and adsorbed NO_2_. The incorporation of Mn enhances the presence of π-bonds in graphite crystallites, oxygen-containing functional groups, and chemisorbed oxygen, which facilitates the oxidation of NO to NO_2_ (eq 9).^64–66^ Owing to its strong chemisorption affinity with carbonaceous surfaces, NO_2_ demonstrates higher stability than NO once adsorbed.^67^ The copresence of NO_2_ and NO enables the accelerated selective catalytic reduction pathway, commonly termed the “fast SCR” reaction, where these gases react with NH_3_ following the Langmuir–Hinshelwood mechanism as shown in eq 10. This pathway markedly enhances de-NO_x_ efficiency since the fast SCR reaction proceeds at a considerably higher rate than the standard SCR reaction.^65,68^

Eley–Rideal mechanism:

8 $$-{\mathrm{NH}}_{\mathrm{2 (ad)}}+{\mathrm{NO}}_{\mathrm{(g)}}\to {\mathrm{NH}}_{2}{\mathrm{NO}}_{\mathrm{(ad)}}\to N_{2\left(g\right)}+H_{2}O_{\left(g\right)}$$

Langmuir–Hinshelwood mechanism:

9 $${\mathrm{NO}}_{\mathrm{(g)}}\overset{M}{\to }{\mathrm{NO}}_{\mathrm{(ad)}}+O_{\mathrm{2 (ad)}}\overset{\mathrm{\pi ‐bonds}}{\to }{\mathrm{NO}}_{\mathrm{2(ad)}}$$

10 $${\mathrm{2NH}}_{\mathrm{3(ad)}}+{\mathrm{NO}}_{\mathrm{(ad)}}+{\mathrm{NO}}_{\mathrm{2(ad)}}\to {\mathrm{2N}}_{2}+{\mathrm{3H}}_{2}O$$

Liu et al.^66^ also observed a reaction mechanism that aligns with the Langmuir–Hinshelwood (L–H) pathway. In their study, a two-step air oxidation method was developed to prepare MnO*_x_*/biochar catalysts for low-temperature SCR of NO: preoxidation at 400 °C enhanced the surface area and introduced acidic functional groups to the biochar, and subsequent postoxidation at 250 °C after Mn impregnation promoted the formation of Mn^4+^ and chemisorbed oxygen species. Within this catalytic system, NH_3_ adsorbs on acidic sites and is activated to NH_2_(ad), while NO adsorbs on Mn sites and is oxidized to NO_2_(ad) by surface-active oxygen. The reaction between adsorbed NH_2_(ad) and NO_2_(ad) then produces N_2_ and H_2_O, achieving a high NO conversion of 97.0% at 150 °C. The mechanism schematic is illustrated in Figure 16.

**Figure 16:**
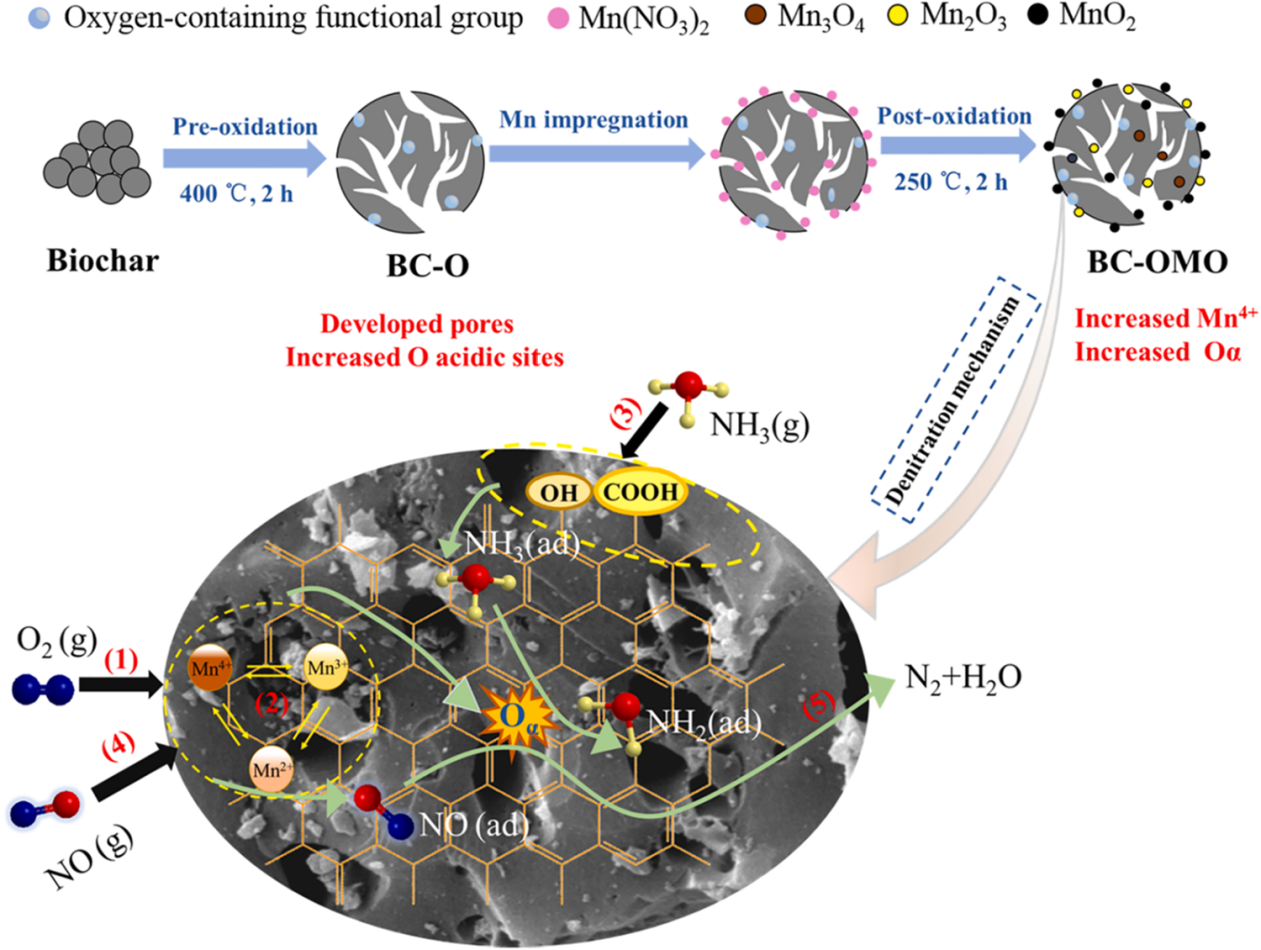
A possible mechanism of the improved denitration performance of MnO*_x_*/biochar catalyst based on air oxidation treatment. (Reprinted with permission from ref 69, Copyright 2021, Elsevier).

### 4.3 Sulfur-Containing Compounds Elimination

Fan et al.^69^ synthesized metal oxide-modified AC derived from walnut shells for flue gas desulfurization, incorporating cobalt, nickel, copper, and vanadium oxides via a blending method. The carbonized walnut shell material was mixed with metal oxide powders at different mass ratios (2%/ 5%/ 7%/ 10%), to which coal tar pitch as the primary binder, along with carboxymethyl cellulose or polyvinyl butyral resin as auxiliary binders, was added. After homogenization in a 70 °C water bath, the mixture was shaped into 3 mm cylindrical pellets using a vacuum extruder and subsequently activated at 900 °C under a CO_2_ flow of 1000 mL/min for 2 h. The resulting metal oxide-loaded carbons displayed enhanced SO_2_ adsorption, attributed to the promotion of basic functional groups on the carbon surface by the incorporated metals. During the high-temperature activation, partial reduction of metal oxides occurred, generating reduced and intermediate valence metal species. These species adsorbed gaseous oxygen and converted it into lattice oxygen, which then reoxidized the metals to their active oxide forms.^70^ The regenerated metal oxides reacted with SO_2_ to form SO_3_, followed by hydrolysis to sulfuric acid. For example, in the V-modified sample, coexisting V^3+^, V^2+^, and V^1+^ species were oxidized by O_2_ to form active V_2_O_5_, which exhibited high catalytic activity toward SO_2_ conversion.^71^ At a 2% metal loading, the SO_2_ adsorption capacities of Co-, Ni-, Cu-, and V-modified carbons increased by 7.7, 19.1, 39.3, and 46.4%, respectively, relative to the unmodified carbon, with the V_2_O_5_-modified variant showing the highest desulfurization performance. The schematic diagram is shown in Figure 17.

**Figure 17:**
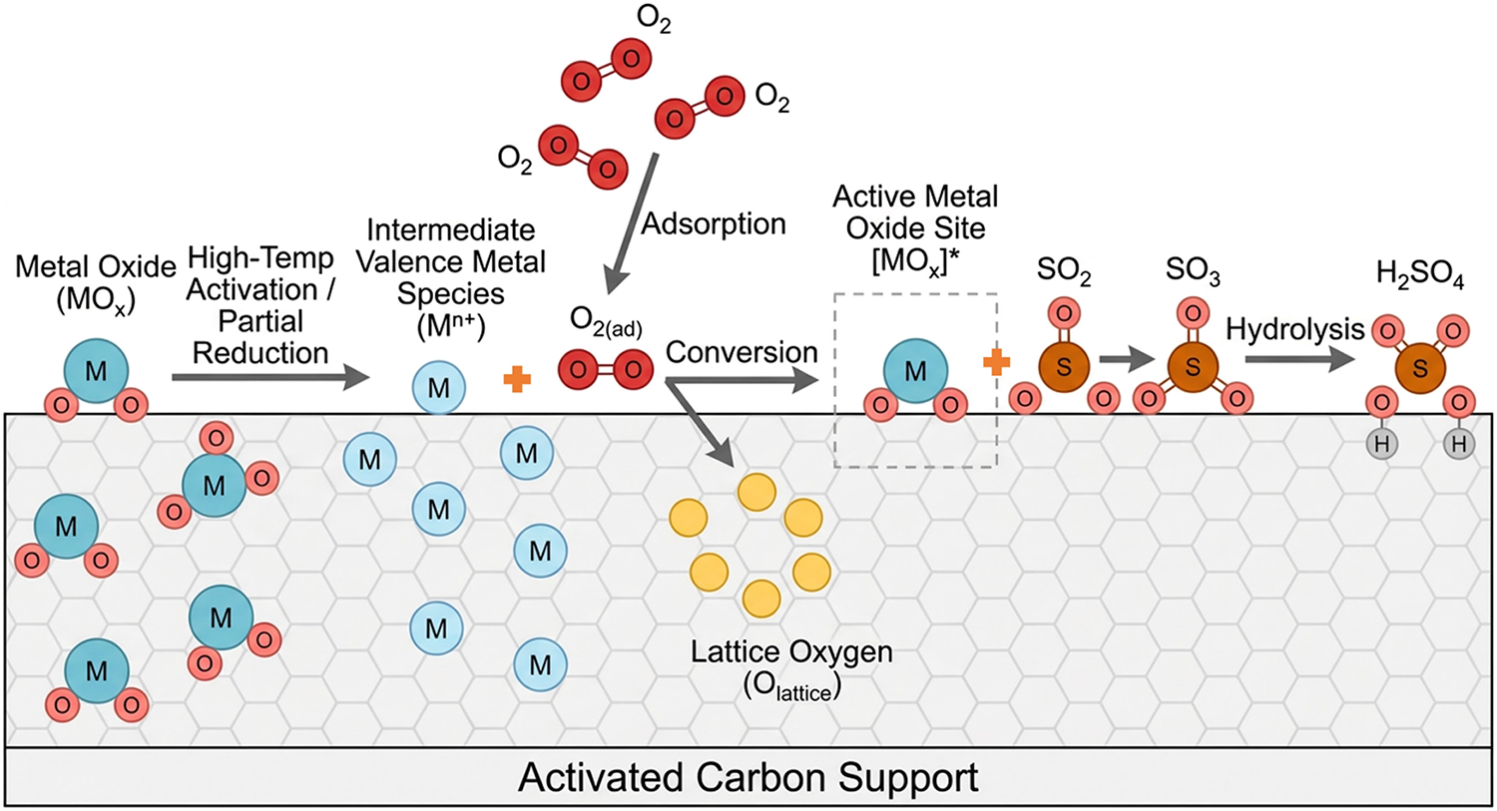
Schematic diagram of the combined elimination of SO_2_ by metal oxides and adsorbed oxygen.

Biochar derived from sewage sludge was modified through impregnation with Ni­(NO_3_)_2_,^72^ resulting in the coexistence of metallic Ni and NiO phases within the carbon matrix alongside an increase in oxygen-containing functional groups. SO_2_ adsorption proceeded through multiple distinct pathways: the adsorbed SO_2_ reacted with surface oxygen species to form NiSO_3_ (eq 11), which subsequently hydrolyzed to yield Ni–H_2_SO_4_ (eq 12). Concurrently, SO_2_ underwent chemisorption on NiO sites, likely forming sulfate species such as C-NiSO_4_ (eq 13). Furthermore, SO_2_ dissolution in the alkaline aqueous layer present on the biochar surface facilitated reactions with inherent mineral components, promoting catalytic oxidation to stable sulfates (eq 14). Notably, the mineral constituents in the biochar were found to contribute 44.6–85.5% of the total SO_2_ adsorption capacity.^73^

11 $$Ni‐‐O+{SO}_{2}\to {\mathrm{NiSO}}_{3}$$

12 $${\mathrm{NiSO}}_{3}+H_{2}O\to \mathrm{Ni}‐‐H_{2}{\mathrm{SO}}_{4}$$

13 $${\mathrm{NiSO}}_{3}+C‐‐O\to C‐‐{\mathrm{NiSO}}_{4}$$

14 $${\mathrm{SO}}_{4}^{2-}+M^{2+}+2H_{2}O\to {\mathrm{MSO}}_{4}\cdot 2H_{2}O\;\left(M=\mathrm{Ca},\mathrm{Ce}...\right)$$

### 4.4 Carbon Dioxides (CO_2_) Elimination

In the development of metal-loaded AC for CO_2_ adsorption, researchers frequently select alkali metals, while certain transition metals have also shown excellent adsorption capacity. Lahuri et al.^20^ prepared wood-derived AC impregnated with metal oxides including CeO_2_, ZnO, and Co_3_O_4_ for CO_2_ capture. The synthesis procedure involved immersing pretreated AC in 0.1 mol/L aqueous solutions of metal salts, followed by agitation at 200 rpm for 8 h. The resulting solids were then filtered and washed with 400 mL of 1% NaHCO_3_ solution, after which they were immersed overnight in 600 mL of fresh 1% NaHCO_3_ solution. Following final filtration, the material was rinsed with distilled water, dried at room temperature for 2 h, and subsequently oven-dried at 110 °C overnight to obtain the metal oxide-loaded adsorbents. Kinetic analysis at 30 °C revealed that the CO_2_ adsorption process followed pseudo-second-order kinetics, indicating a chemisorption-dominated mechanism. The CeO_2_-modified carbon exhibited alkaline surface properties where surface oxygen species chemically reacted with CO_2_ to form carbonate complexes (eq 15). Experimental results demonstrated that CeO_2_/AC achieved the highest CO_2_ adsorption capacity of 52.78 mg/g, reaching equilibrium within 10 min.

15 $$CO_{2}\;\left(\mathrm{adsorbate}\right)+O\;\left({\mathrm{surface of CeO}}_{2}\right)\to \left[{\mathrm{CO}}_{2}‐‐O\right]\to {\mathrm{CO}}_{3}\;\left(\mathrm{chemisorption}\right)$$

### 4.5 Mercury (Hg^0^) Elimination

Liu et al.^74^ developed biochar from rice straw through eutectic salt synthesis using K_2_CO_3_ and Li_2_CO_3_, followed by modification with copper sulfide. At 100 °C and in the presence of Cu^2+^ ions, the maximum cumulative mercury adsorption capacity reached 838 μg/g over 600 min. The adsorption mechanism followed the Mars-Maessen pathway, where S^2–^ species served as primary adsorption sites while Cu^2+^ ions oxidized adsorbed elemental mercury to Hg^2+^ species, as shown in eqs 16 and 17. These oxidized mercury species then reacted with S^2–^ to form stable HgS, as represented in eq 18, with simultaneous reduction of Cu^2+^ to Cu^+^ species.

16 $${\mathrm{Hg}}_{g}^{0}+\mathrm{surface}\to {\mathrm{Hg}}_{\mathrm{ad}}^{0}$$

17 $${\mathrm{Hg}}_{\mathrm{ad}}^{0}+2{\mathrm{Cu}}^{2+}\to 2{\mathrm{Cu}}^{+}+{\mathrm{Hg}}_{\mathrm{ad}}^{2+}$$

18 $${\mathrm{Hg}}_{\mathrm{ad}}^{2+}+S^{2‐}\to \mathrm{HgS}$$

The Mars-Maessen mechanism^75,76^ comprises two critical stages in the catalytic process. In the initial oxidation stage, gaseous reactants such as elemental mercury adsorb onto the catalyst surface and subsequently react with lattice oxygen derived from metal oxides including manganese oxides and chromium oxides. This interaction yields oxidized products like mercury oxide, as represented in eq 19, while simultaneously consuming lattice oxygen and reducing the local catalyst structure through decreased metal valence states. In the subsequent regeneration stage, gaseous oxygen molecules adsorb onto the reduced catalyst surface, replenishing the depleted lattice oxygen and restoring the catalyst’s oxidative capacity, completing the catalytic cycle as shown in eq 20.

19 $${\mathrm{Hg}}^{0}+M^{n+}O\to \mathrm{HgO}+M^{\left(n‐2\right)+}$$

20 $$2M^{\left(n‐2\right)+}+O_{2}\to 2M^{n+}O$$

Similar mechanism has been proposed by Shan et al.^49^ This study reports the preparation of a novel manganese–iron modified magnetic biochar adsorbent (MnFe4%(3/10)/CSWU700) using cotton straw as the feedstock through microwave activation and ultrasound-assisted impregnation. The adsorbent demonstrated a high Hg^0^ removal efficiency of 87.1% and an adsorption capacity of 531.9 μg/g in simulated flue gas at 120 °C. Based on characterization results from XPS, BET, and XRD, the proposed Hg^0^ removal mechanism can be reasonably deduced as follows: Hg^0^ is first adsorbed onto the well-developed pore structures and oxygen-containing functional groups created by microwave activation, and then oxidized by highly dispersed Mn–Fe mixed oxides like MnO_2_ and Fe_3_O_4_ to form HgO. Part of the Hg^0^ may also react with NO_2_ or SO_3_ in the flue gas to form Hg­(NO_3_)_2_ or HgSO_4_. During this process, chemisorbed oxygen (O_β_) and lattice oxygen participate in the oxidation reaction and are consumed, while gaseous O_2_ in the flue gas can replenish the active oxygen species, sustaining the adsorption-oxidation cycle. The study also found that the Mn–Fe bimetallic adsorbent treated with ultrasound significantly outperformed single-metal or nonultrasonicated samples, which is attributed to the optimized dispersion of active components and the formation of a more abundant pore structure facilitated by ultrasonic treatment. A schematic diagram of the mechanism can be found in Figure 18.

**Figure 18:**
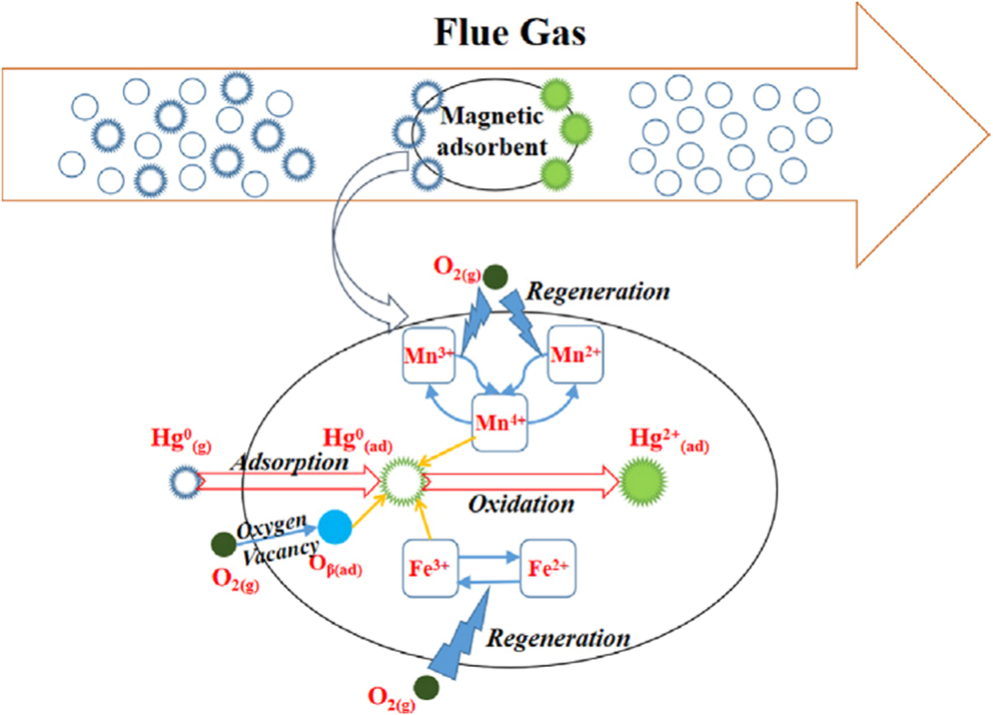
Proposed Hg^0^ removal mechanism of Mn–Fe modified magnetic biochar adsorbent. (Reprinted with permission from ref 49, Copyright 2019, Elsevier).

In addition, Xiang et al.^22^ showed that impregnating AC with salts like MnCl_2_ or CoCl_2_ leverages the inherent (*n*–1) d^1–10^ns^2^ valence electron configuration of the transition metals, whose hybrid orbitals facilitate redox reactions with Hg^0^. According to Qin et al.,^77^ chemisorption sites and oxidation sites, corresponding to electron-rich oxygen vacancies and species including Fe^3+^, lattice oxygen, and chemisorbed oxygen respectively, lead to the formation of Hg–O–Fe-O_*x*–1_ and Hg-OM complexes. Wen et al.^1^ identified that chlorinated carbon groups, metal oxide species, and sulfur-containing functional groups all provide active sites for mercury capture.

### 4.6 Chemical Warfare Agents (CWAs) Elimination

In 1924, R.E. Wilson and J.C. Whetzel^78^ developed the first copper-impregnated AC using an ammoniacal copper carbonate solution for gas mask filters. This material, later designated as “Whetlerite”,^79^ was significantly enhanced during World War II with the introduction of the ASC variant impregnated with copper, chromium, and silver. This improved formulation proved highly effective against toxic small molecules including phosgene (COCl_2_), hydrogen cyanide (HCN), cyanogen chloride (ClCN), and arsine (AsH_3_).^80^ Due to chromium’s recognized carcinogenicity and environmental impact, subsequent research focused on replacing chromium with alternative metals such as molybdenum, zinc, and vanadium. In these systems, copper, vanadium, and molybdenum ions primarily target hydrogen cyanide and cyanogen chloride, while silver ions specialize in arsine adsorption. Whetlerite materials are susceptible to aging,^81^ a process where moisture and carbon dioxide adsorption gradually diminishes their activity under ambient conditions. To address this limitation, triethylenediamine (TEDA) has been incorporated as an antiaging additive, helping maintain adsorption capacity and prevent metal ion deactivation during storage under high temperature and humidity.

Tolles et al.^82^ prepared ASMT/ASVT-type Whetlerite by impregnating AC particles with salt solutions containing copper (7–15%), silver (0.03–0.1%), and either molybdenum or vanadium (2–4%), followed by the addition of tartaric acid (below 8%). The manufacturing process involved sequential low-temperature drying (225–275 °F), high-temperature heat treatment (350–600 °F), and final spraying with triethylenediamine (TEDA, 2–6%) before drying. Their investigation revealed that both molybdenum and vanadium, when combined with copper, effectively prevented cyanogen gas ((CN)_2_) leakage. Vanadium-impregnated AC demonstrated superior hydrogen cyanide adsorption performance compared to molybdenum-impregnated samples. Products containing vanadium and 6% TEDA achieved hydrogen cyanide breakthrough times of 45–60 min, whereas copper-containing samples showed breakthrough times of approximately 30 min.

Coconut-shell AC decorated with Cu (15–20 wt %), Zr (2–8 wt %), and minor amounts (0.1–5 wt % each) of Ce, W and V was synthesized by Zhao et al.^83^ via incipient-wetness impregnation to confer protection against HCN and CNCl. The protocol comprised dissolution of stoichiometric metal salts in an aqueous-ammonia mixture at 60–80 °C under vigorous agitation, followed by dropwise addition of the resulting solution to the carbon support with continuous stirring. After complete uptake, the slurry was hermetically sealed and aged for 1–4 h, then activated under a controlled hot-air stream that was ramped to 100–160 °C and maintained for 24 h.

The removal mechanisms of different CWAs by metal-loaded AC have been revealed in previous research. Cu and Cr can act as catalyzers or participate in hydrolysis reaction of HCN and ClCN (eqs 21–23 and eqs 24–25) in the moist air.^84^

21 $$4\mathrm{HCN + 2CuO}\to 2\mathrm{Cu}{\left(\mathrm{CN}\right)}_{2}+2H_{2}O$$

22 $$\mathrm{2Cu}{\left(\mathrm{CN}\right)}_{2}\to 2{\mathrm{CuCN + C}}_{2}N_{2}$$

23 $$C_{2}N_{2}+2H_{2}O\to {{\mathrm{(CONH}}_{2})}_{2}$$

24 $${\mathrm{CNCl + H}}_{2}O\overset{{\mathrm{Cu}}^{2+}}{\underset{\mathrm{Cat}.}{\to }}\mathrm{HOCN}+\mathrm{HCl}$$

25 $$HOCN+H_{2}O\to {\mathrm{NH}}_{3}+{\mathrm{CO}}_{2}$$

## 5 Challenges and Future Perspectives

### 5.1 The Knowledge Gap in Adsorption Mechanisms

The current mechanistic framework for gas elimination over transition metal-modified ACs is built upon multiple, often coexisting models—including physisorption/chemisorption,^26^ Mars-Maessen redox cycles, Eley–Rideal^63,64^ or Langmuir–Hinshelwood^64–66^ pathways for SCR,^65,68^ and hydrolysis routes for chemical agents. These models primarily rationalize performance from the analysis of static end points (e.g., HgS, sulfates, N_2_, or hydrolysis products). This reliance on postreaction analysis, rather than direct observation of dynamic processes, leaves a series of intertwined fundamental questions unresolved, hindering the development of a unified and predictive mechanistic picture.

A primary ambiguity concerns the functional demarcation between metal centers and the carbon support. It remains unclear whether metal species like oxides or ions of Cu, Mn, Fe function predominantly as direct chemisorption sites for gases like Hg^0^ or HCN, or primarily as catalytic centers that activate key reactants like O_2_, H_2_O, lattice oxygen for subsequent oxidation or hydrolysis. Concurrently, the role of the carbon matrix is debated, specifically whether it acts as a passive, high-surface-area scaffold or participates actively by forming distinct C–O–M interfacial sites through its functional groups and defects, thereby mediating critical electron transfer processes in redox cycles. The prevailing mechanistic models are often constructed through mutual corroboration among fundamental surface chemistry, solid-state physics, and computational chemistry,^85,86^ rather than being directly observed.

Further complexities arise in multicomponent and multipollutant systems. For materials designed for the simultaneous removal of SO_2_, NO*_x_*, and Hg^0^, the nature of the observed synergy is ambiguous. It is not established whether the enhancement stems from a simple juxtaposition of independent functions or from more sophisticated, coupled reaction pathways enabled by electron transfer across metal–metal or metal–carbon interfaces. Moreover, the molecular-scale origins of deactivation in realistic, complex environments represent a significant “black box.” The specific mechanisms—such as whether SO_2_ or H_2_O selectively poison active metal sites via chemical bonding or primarily cause physical blockage of the carbon pore network—are poorly defined.

In essence, prevailing mechanistic explanations are largely self-consistent interpretations of macroscopic performance data, inferred from static snapshots before and after reaction. The inability to directly probe the dynamic evolution of active sites, intermediates, and interfacial processes in operando constitutes the core knowledge gap. This fundamental limitation precludes a truly rational, atomically informed design strategy for next-generation metal-loaded AC adsorbents and catalysts.

### 5.2 Deficiency in Operando Characterization

The scientific ambiguities in mechanism stem from a critical technical bottleneck: the heavy reliance on ex situ characterization methodologies. These methods are inherently incapable of capturing the real-time, dynamic evolution of catalysts under working conditions. The inability to directly monitor changes in oxidation states, coordination environments, surface intermediates, and gaseous products in real-time forces mechanistic understanding to rely on inference. Therefore, a paradigm shift toward operando (in situ under working conditions) characterization is not merely beneficial but essential to transform mechanistic studies from indirect correlation to direct observation. Emerging integrated platforms exemplify this shift. For example, coupling operando spectroscopic techniques like DRIFTS^87,88^ with online mass spectrometry (MS) enables the molecular-level, time-resolved tracking of surface species and their evolution. Looking ahead, the deployment of even more advanced operando platforms—synergistically integrating high spatiotemporal-resolution synchrotron techniques, multistimuli electron microscopy, and real-time product analysis—will empower researchers to directly, quantitatively, and correlatively resolve the dynamic interplay among catalyst structure, surface chemistry, and performance at the atomic/molecular scale.^89^ Only by bridging this technical gap can the intrinsic “structure-performance” relationship be truly revealed, closing the current mechanistic knowledge gaps.

### 5.3 Engineering Bottlenecks in Metal-Loading Processes for Industrial Scale-Up

The current research focus on lab-scale synthesis optimization (e.g., incipient wetness impregnation,^21,84^ sol–gel methods^27,61^ ) encounters a significant translational gap when moving toward industrial application. Batch processes face major challenges in ensuring uniform dispersion of metal precursors and reproducibility across large volumes of activated carbon supports, often leading to local metal aggregation and pore blockage. Moreover, complex, multistep synthesis protocols are frequently time- and energy-intensive, and may rely on expensive or hazardous precursors, compromising economic viability and green chemistry principles. A critical, yet often overlooked, engineering challenge is the formulation of high-performance powder materials into robust structures suitable for industrial fixed- or moving-bed reactors, which require manageable pressure drop and resistance to attrition. To bridge this lab-to-plant divide, future development must parallel materials innovation with process engineering. This entails exploring continuous, modular production flows, such as integrated flow reactor systems for consistent metal deposition and washing,^90^ to enhance throughput and uniformity. Concurrently, efforts must be directed at designing structured adsorbents with high mechanical integrity, such as spherical pellets, monoliths, or wash-coated honeycombs, that preserve high surface area and site accessibility while meeting practical reactor demands.^91^ Furthermore, employing precision loading techniques like Atomic Layer Deposition (ALD)^92^ or strategies to engineer Strong Metal–Support Interactions (SMSI) is a crucial forward-looking approach to stabilize metallic nanoparticles against sintering and leaching, thereby addressing deactivation under harsh operating conditions.

## 6 Conclusion

This review provides a comprehensive overview of the evolving landscape of transition metal-modified ACs as highly effective materials for hazardous gas elimination. The discussed preparation strategies—ranging from conventional impregnation and doping to advanced sol–gel and composite approaches—enable precise control over metal dispersion and interfacial synergies. Mechanistic analyses reveal the interplay between physisorption, driven by porous architecture, and chemisorption, facilitated by redox-active metal sites and tunable surface functional groups, underscoring the multifaceted roles of structural, chemical, and operational factors in governing adsorption performance. Applications across a broad spectrum of pollutants, including VOCs, NO_*x*_, SO_2_, Hg^0^, CO_2_, and CWAs, demonstrate the versatility of these materials in catalytic oxidation, selective reduction, and reactive capture, often achieving superior efficiencies in complex flue gas or ambient conditions. Notwithstanding persistent challenges—such as pore occlusion from excessive metal loading, deactivation under humid or multipollutant environments, and regeneration limitations—emerging trends informed by mechanistic insights hold immense promise for overcoming these barriers. Looking ahead, future progress hinges on bridging the fundamental mechanistic gap through operando characterization and overcoming scale-up bottlenecks via engineered synthesis and structured adsorbent design. Ultimately, transition metal-modified ACs emerge as robust, economically viable platforms poised to significantly contribute to global efforts in air pollution mitigation and environmental sustainability.
